# Functional characterization of *BC039389-GATM* and *KLK4-KRSP1* chimeric read-through transcripts which are up-regulated in renal cell cancer

**DOI:** 10.1186/s12864-015-1446-z

**Published:** 2015-03-27

**Authors:** Dorothee Pflueger, Christiane Mittmann, Silvia Dehler, Mark A Rubin, Holger Moch, Peter Schraml

**Affiliations:** Institute of Surgical Pathology, University Hospital Zurich, Zurich, Switzerland; Competence Center Personalized Medicine, ETH and University of Zurich, Zurich, Switzerland; Cancer Registry Zurich and Zug, University Hospital Zurich, Zurich, Switzerland; Institute of Precision Medicine of Weill Cornell Medical College and New York-Presbyterian Hospital, New York, USA; Department of Pathology and Laboratory Medicine, Weill Cornell Medical College, New York, USA

**Keywords:** RNA-Seq, Renal cell carcinoma, Read-through, *GATM*, *KLK4*, Chimera

## Abstract

**Background:**

Chimeric read-through RNAs are transcripts originating from two directly adjacent genes (<10 kb) on the same DNA strand. Although they are found in next-generation whole transcriptome sequencing (RNA-Seq) data on a regular basis, investigating them further has usually been refrained from. Therefore, their expression patterns or functions in general, and in oncogenesis in particular, are poorly understood.

**Results:**

We used paired-end RNA-Seq and a specifically designed computational data analysis pipeline (FusionSeq) to nominate read-through events in a small discovery set of renal cell carcinomas (RCC) and confirmed them in a larger validation cohort.

324 read-through events were called overall; 22/27 (81%) selected nominees passed validation with conventional PCR and were sequenced at the junction region. We frequently identified various isoforms of a given read-through event. 2/22 read-throughs were up-regulated: *BC039389-GATM* was higher expressed in RCC compared to benign adjacent kidney; *KLK4-KRSP1* was expressed in 46/169 (27%) RCCs, but rarely in normal tissue. *KLK4-KRSP1* expression was associated with worse clinical outcome in the patient cohort. In cell lines, both read-throughs influenced molecular mechanisms (i.e. target gene expression or migration/invasion) in a way that counteracted the effect of the respective parent transcript *GATM* or *KLK4*.

**Conclusions:**

Our data suggests that the up-regulation of read-through RNA chimeras in tumors is not random but causes regulatory effects on cellular mechanisms and may impact patient survival.

**Electronic supplementary material:**

The online version of this article (doi:10.1186/s12864-015-1446-z) contains supplementary material, which is available to authorized users.

## Background

With the launching of large consortium projects like ENCODE or FANTOM, the research community has become highly aware of an underestimated complexity of the eukaryotic genome and transcriptome. The “one gene - one RNA - one protein” paradigm has been abandoned by realizing that coding and non-coding RNA is exerting multiple functions in regulating cellular systems [1]. New technologies were utilized on sequencing the transcriptome to identify new classes of RNAs, for example chimeric RNA molecules containing exons from two directly adjacent genes of the same transcriptional orientation, termed read-through RNAs. They supposedly arise through mechanisms not a priori requiring genomic rearrangements [2-4]. In fact, read-throughs favorably occur at minimal intergenic distance between the parent genes (approx. 10 kb) [5]. Several years ago came the first reports of systematic large-scale computational analyses of read-through RNAs which were termed “Transcription-mediated gene fusion”, “Tandem chimerism” and “Conjoined genes” [5-7]. The authors of these studies used deposited data from EST data bases and confirmed their predictions only in normal human tissues and a few cell lines. One can find only two recent comprehensive read-through studies in tumors, one on breast cancer [8] and one on prostate cancer [9]. Two additional studies in cancer report on fusion transcripts/chimeric RNAs of which the vast majority is suspected of being read-throughs, although this is not clearly documented [10,11]. In other RNA-Seq papers, read-throughs are mentioned as a side note or in the supplement as they were not the focus of interest [2,12,13]. The only comprehensive study on renal cancer employing RNA-Seq has explicitly excluded read-throughs from the list of RNA fusions [14]. RNA read-through formation seems to be evolutionary conserved. A study in Arabidopsis thaliana demonstrated evidence, that depletion of RNA binding proteins causes impairement of transcription termination, thereby enhancing the expression of RNA read-throughs which in parallel influence the expression levels of their parent genes [15]. Little is known about the biological or disease-associated function of read-through RNAs in humans. Some read-throughs might encode functional chimeric proteins as exemplified in the case of TWE-PRIL [16] or LY75-CD302 [17]. *SLC45A3-ELK4* as the prototype read-through in prostate cancer is not just a biomarker [2,3] but has been shown to induce prostate cancer proliferation in-vitro in a recent study by Zhang et al. [4]. The same group also demonstrated that *SLC45A3-ELK4* is generated by cis-splicing and that its formation is mechanistically intertwined with androgen signaling. In summary, chimeric read-through transcripts may have implications in carcinogenesis.

Here, we explore RNA read-throughs by sequencing the transcriptome of human renal cell carcinoma (RCC), a malignancy where nothing major is known on read-through expression yet, and elaborate on the potential functions of two examples relevant to renal carcinogenesis.

## Results

### Numerous read-through RNA chimeras are expressed in RCC

**Figure 1:**
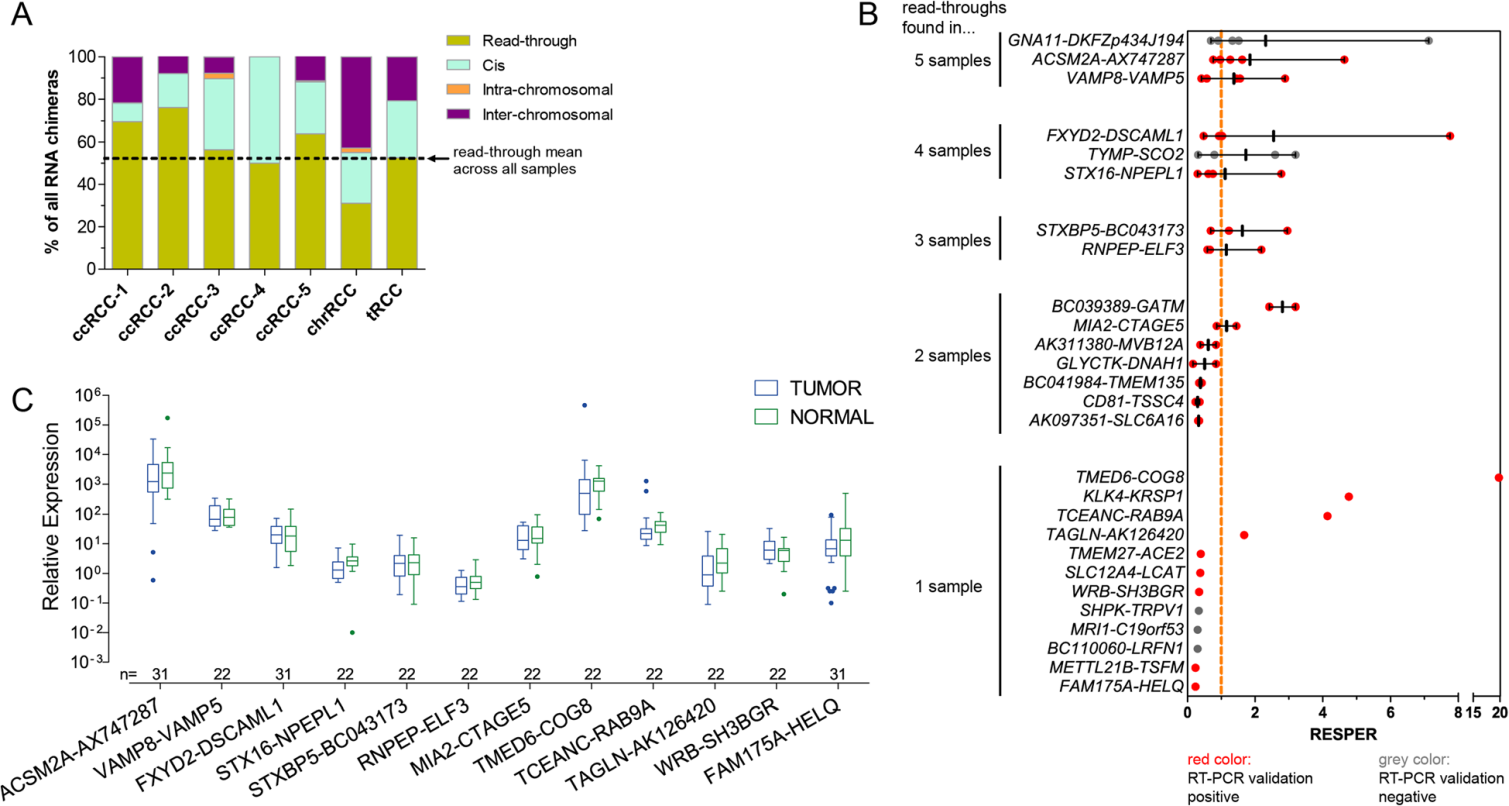
A panel of read-throughs is expressed in RCC. (**A**) The fraction of different classes of RNA chimeras called by FusionSeq from the RNASeq data in seven frozen RCC samples. The clinical characteristics of the samples are given elsewhere [30]. ccRCC, clear cell RCC; chrRCC, chromophobe RCC; tRCC, Xp11 translocation RCC. (**B**) 27 read-throughs which were chosen for further validation by reverse transcription (RT)-PCR. Plotted are the individual RESPER scores per sample and the mean RESPER with range if the read-through was found in more than 1 sample. (**C**) Quantitative evaluation of 12 read-throughs by TaqMan assays in frozen tissue of RCC and matched adjacent normal kidney, plotted as Tukey boxplots. No read-through has significant differential expression between tumor and normal tissue as calculated by student’s *t*-test with correction for multiple testing by Bonferroni method. The blue outlier dot in *TMED6-COG8* is the tRCC whose detailed analysis is featured elsewhere [30].

The RNA-Seq analysis by FusionSeq called 324 read-throughs across the sample set representing about half (mean of 52.3%) of all RNA chimera calls (Figure 1A & Additional file 1: Figure S1). Most of them had low (≤2) RESPER (Ratio of empirically computed supportive paired-end reads) values which is interpretable as humble expression levels of most read-throughs. RESPER not only is a confidence score for the candidate call by the software, it also gives an estimate about the expression level of the chimeric transcript. Due to limited availability of RCC tissue we selected an arbitrary number of top- (RESPER > 2 (n = 13)) and bottom- (RESPER < 0.4 (n = 14)) candidates and confirmed 11 of 13 (85%) top- and 11 of 14 (79%) bottom-candidates with conventional reverse transcription (RT)-PCR (Figure 1B & Additional file 1: Figure S2). Based on this finding, we assume that candidates with RESPER between 0.4 and 2 also have a true positive rate around 79-85%. Sanger Sequencing of the PCR products enabled us to determine the read-throughs’ junction sequence and exon composition around this region (Table 1). Most read-through events (13 of 22) generated two to five different isoforms. For nine read-throughs existed only a single transcript. The most obvious splicing pattern (53% of isoforms) is the exclusion of terminal exons from the upstream parent gene and initial exons from the downstream parent gene, using known exon-intron boundaries. Other isoforms (39%) used new GT—AG splice sites in introns or exons to lengthen or shorten an exon or to introduce a new exon from intergenic sequence. A third group of isoforms (10%) retained intergenic sequence, in some instances suggesting that the 3′ parent contributes an extended 3′UTR to the 5′ parent gene. Knowing the read-throughs’ exon compositions enabled us to build putative coding sequences. Only in 12% of the isoforms the exon junction was in-frame which might fuse both parent open reading frames (ORFs) forming an intact fusion ORF (Table 2). Most often, exon junctions were outside of the 5′ parent’s ORF (31%) or caused frameshifts and premature stop codons in the 5′ genes (35%). About 20% of the isoforms were originating from read-throughs between known genes and non-coding RNAs, merely annotated with data bank accession numbers, and therefore termed “non-classical”. The functional consequence in such instances is unclear. One read-through was a known antisense transcript [11].

**Table 1:** **Characteristics of 22 confirmed read-throughs in RCC**

**Read-through**	**Type**	**Transcript isoforms**		**Exon junction**	**Exclusion terminal/initial exons**	**Modification/Introduction exon**	**Retention intergenic sequence**	**GT-AG splice site**	**Supporting EST**	**Coding consequence**	**TaqMan**
MIA2-CTAGE5	classical	1		M6middle-C4		x		y	N	1	x
WRB-SH3BGR	classical	2		W4-S3	x			y	Y	1	x
				W4-S3 (S6-7 out)	x			y		1	x
FAM175A-HELQ	classical	1		F8-H3	x			y	Y	1	x
TCEA NC-RAB9A	classical	2		T4-R2	x			y	N	2	x
				T4-R2	x			y		2	x
FXYD2-DSCAML1	classical	5		F3e-D2	x			y	N	2	x
				(F3c out) F3e-D2	x			y	N	2	x
				F3e-D2 (D2 intra-spliced)		x		y	N	2	x
				(F3c out) F3e-D2 (D2 intra-spliced)		x		y	N	2	x
				F3e-intergenic seq-D2			x	n	N	2	x
SLC12A4-LCAT	classical	1		S26middle-L2		x		y	N	2	
CD81-TSSC4	classical	1		C8middle-T1		x		y	N	2	
VAMP8-VAMP5	classical	3		V3middle-V2		x		n	Y	2	x
				(V3 intra-spliced) V3middle-V2		x		n	Y	1	x
				V3middle2-V2		x		n	Y	2	x
STX16-NPEPL1	classical	4		S8-N2a (N3 out)	x			y	Y	1	x
				S8-intergenic ex on 2-N2a (N3 out)		x		y	Y	3; fsX42	
				S8-intergenic ex ons 1,2-N2b (N3 out)		x		y	Y	3; fsX92	
				S8-N2b (N3 out)	x			y	Y	3; fsX42	
TMED6-COG8 ^*^	classical	3		T1-C2	x			y	Y	3; fsX17	x
				T2-C2	x			y	Y	3; fsX4	x
				T3-C2	x			y	Y	3; fsX17	
TMEM27-ACE2	classical	4		T5-A2	x			y	N	3; fsX9	
				T5-A2middle		x		y	N	3; fsX16	
				T4-A2	x			y	N	3; fsX9	
				T4-A2middle		x		y	N	3; fsX16	
METTL21B-TSFM	classical	1		M2-T2 (T5 out)	x			y	Y	3; fsX105	
GLYCTK-DNAH1	classical	3		(G2 out) G3-D1middle		x		y	N	3; fsX14	
				G3-intergenic seq-D1middle(D1c in)		x	x	y	N	3; fsX1	
				G3-D1middle		x		y	N	3; fsX14	
AKO97351-SLC6A 16	classical	5		A1-S2	x			y	N	3; fsX23	
				A2-S2	x			y	N	3; fsX22	
				A3middle-S2		x		y	N	2	
				A1-S8	x			y	N	3; fsX7	
				A1-S9	x			y	N	3; fsX2	
KLK4-KRSP1	not classical	5	KKv1	K3-K4	x			y	Y	4; fsX56	x
			KKv2	K5middle-K4		x		n	Y	4; fsX3	x
			KKv3	K5middle2-K4		x		y	Y	4; fsX56	
			KKv4	K1-K4	x			y	Y	4; fsX56	
			KKv5	K1-K3	x			y	Y	4; fsX72	
BC039389-GA TM	not classical	2	BGv1	B3-G2	x			n	Y	4	x
			BGv2	B3-G3	x			y	Y	4	x
AK311380-MVB12A	not classical	2		A2-M2	x			y	Y	4	
				(A1 intra-spliced) A2-M2	x			y	Y	4	
BC041984-TMEM135	not classical	1		B2-T2	x			y	N	4	
RNPEP-ELF3	not classical	1		Ri6middlie-E1middle		x		y	Y	5	x
ACSM2A-AX747287	extended 3′ UTR	2		A 15-intergenic seq-A			x		N	2	x
				A 15middle-Amiddle		x		n	N	2	
TAGLN-AK126420	extended 3′ UTR	1		T5-intergenic seq-A			x		Y	2	x
STXBP5-BC043173	extended 3′ UTR	1		S 28-intergenic seq-B			x		y	2	x
**TOTAL: 22**		**51**			**27 (53%)**	**20 (39%)**	**5 (10%)**				

Read-throughs are sorted in three types: classical, if the parent genes’ structure and ORF are well defined; non-classical, if at least one parent gene is incompletely defined non-coding RNA or pseudogene or the read-through being an antisense transcript; extended 3′UTR, if the 3′ parent likely represents a longer 3′UTR sequence to complement the 5′ parent with. The number of isoforms per read-through is given in the third column, together with the names of *KLK4-KRSP1* and *BC039389-GATM* isoforms specifically featured in this publication (labeled red). The exon junction is given between 5′ parent and 3′ parent gene according to their NCBI entries and hg19 assembly, as exemplified with *WRB-SH3BGR*: *WRB* exon 4 (W4) is spliced to *SH3BGR* exon 3 (S3). A second isoform with same exon junction exists where *SH3BGR* exons 6 and 7 are spliced out (S6-7 out). Some exons were shortened at previously unknown positions (termed middle, or middle2 if different positions were used) before they were connected to the other parent gene. Some exons were spliced to lose a short part of the sequence inside the exon (intra-spliced). Some isoforms retained the entire or partial intergenic sequence (seq) between the parent genes or spliced elements of it (exons). An exon junction with intronic sequence is indicated as “i” (for example, Ri6middle = shortened intron 6 of *RNPEP*)). The second to last column shows the coding consequence of the exon junction further described in Table 2. The last column “TaqMan” indicates the isoforms that were targeted in the expression screening depicted in Figure 1C. *TMED6-COG8 has been identified as part of this study but evaluated in detail elsewhere [30].

**Table 2:** **Coding consequence of the read-through isoforms**

**Coding consequence**	**n**	**% Isoforms**	**Functional consequence**
1 = exon junction in-frame	6	11.8%	Predicted fusion protein ORF
2 = exon junction outside of ORF	16	31.4%	5′partner fully conserved, 3′partner fully or partially conserved OR regulatory OR NMD
3 = exon junction frameshift, premature stop codon	18	35.3%	5′partner partially conserved, 3′partner fully or partially conserved OR regulatory OR NMD
4 = exon junction with non-coding RNA	10	19.6%	Encoding partner partially conserved OR regulatory OR NMD
5 = antisense transcript	1	2.0%	Regulatory
**TOTAL**	51		

The numbering in column 1 is referring to the second to last column of Table 1. NMD, Nonsense-mediated decay; ORF, open reading frame.

In this data set, Fusion Seq called many read-throughs only in one or two samples (Figure 1B). However, their expression was mostly not restricted to just one sample as shown in subsequent quantitative measurement of read-through expression in a larger sample set. We selected 14 read-throughs (Mean RESPER > 1 in Figure 1B and/or encoding putative fusion protein) for differential expression analysis by TaqMan qPCR and found that 12 were expressed in all RCC samples (this cohort comprising one Xp11 translocation RCC (tRCC), one chromophobe RCC (chrRCC), four papillary RCCs (pRCC), 26 clear cell RCCs (ccRCC)) and at levels that were equal to their matched benign kidney tissues (Figure 1C). However, two read-throughs presented with exceptional expression pattern.

### Read-throughs *BC039389-GATM* and *KLK4-KRSP1* are overexpressed in RCC

**Figure 2:**
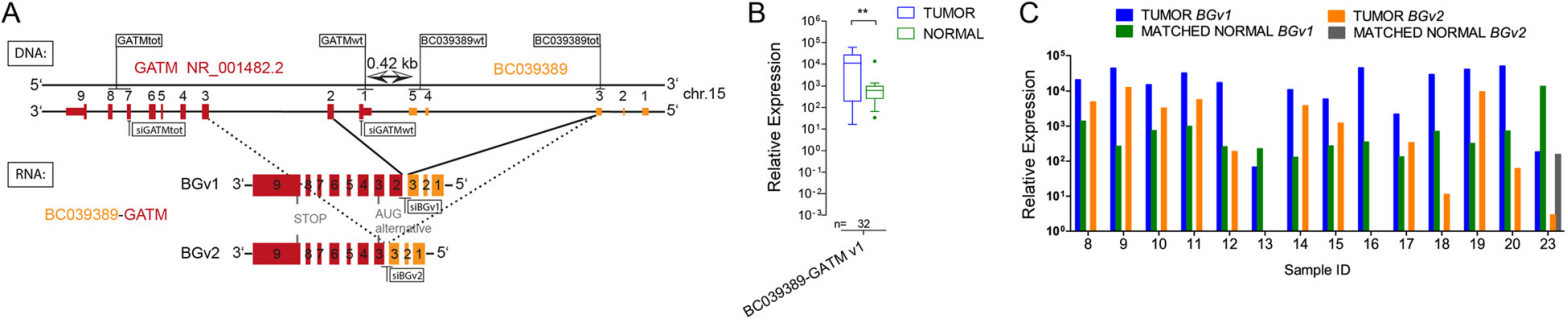
Description of read-through *BC039389-GATM.* (**A**) DNA: Genomic structure of *BC039389* and *GATM* on the DNA (−) strand. RNA: Two isoforms are expressed from the genomic locus both utilizing the same alternative ATG in *GATM* exon 3 for putative protein translation. TaqMan assay positions and the positions targeted by siRNAs are indicated. (**B**) Differential expression of *BC039389-GATM* isoform 1 (*BGv1*) in tumor vs. normal kidney tissue (**p < 0.01, student’s *t*-test with Bonferroni correction). (**C**) Comparison of *BGv1* and *BGv2* expression in 14 frozen tumor/normal sample pairs using TaqMan assays.

The first read-through which caught our interest was *BC039389-GATM* (*BG*) occurring between the non-coding RNA *BC039389* with unknown function and *GATM* (Glycine amidinotransferase; alias AGAT) (Figure 2A). Alternative splicing of *GATM* exons 2 or 3 produces the two *BC039389-GATM* isoforms v1 and v2. *BGv1* was expressed in all RCC samples and the levels were significantly elevated compared to the matched normal kidney tissues (Figure 2B). A direct comparison in 14 samples revealed that the levels of the minor *BC039389-GATM* variant (*BGv2*) were one log lower in most cancer tissues and not detectable in most normal tissues (Figure 2C).

**Figure 3:**
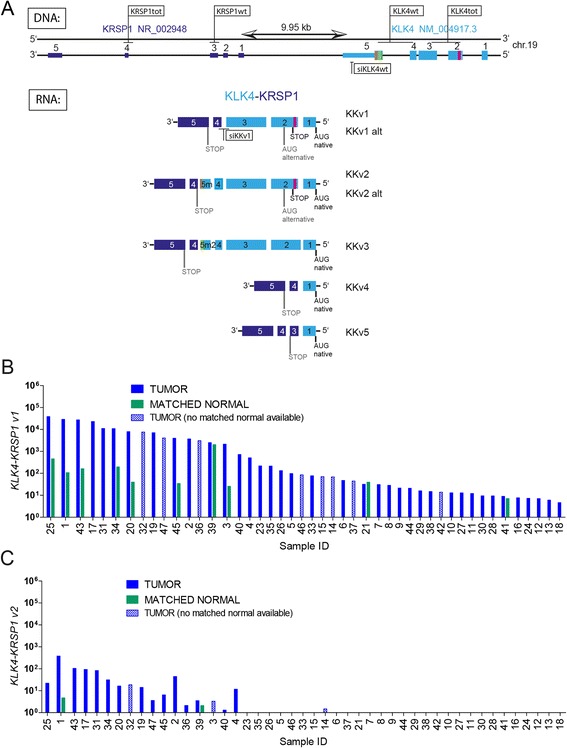
Description of read-through *KLK4-KRSP1.* (**A**) DNA: Genomic structure of *KLK4* and *KRSP1* on the DNA (−) strand. RNA: At least five isoforms are expressed from the genomic locus. A pink line represents the splice site in *KLK4* exon 2 which leads to a frameshift and subsequent STOP codon in the RNA and the use of an alternative (alt) ATG START codon in putative KKv1 and KKv2 proteins. Green and brown lines indicate the splice sites in *KLK4* exon 5 (5 m = exon 5 middle, 5 m2 = exon 5 middle with different splice site) utilized in *KKv2* and *KKv3*, respectively. TaqMan assay positions and the positions targeted by siRNAs are indicated. (**B**) Expression levels of *KLK4-KRSP1* isoform 1 (*KKv1*) in 46 frozen tumor/normal sample pairs using TaqMan assay. (**C**) *KKv2* expression in tumor/normal tissue pairs was screend for 46 *KKv1*-positive samples.

The second interesting read-through was *KLK4-KRSP1* (*KK*) involving *KLK4*, a member of the serine protease family of tissue kallikreins and its pseudogene *KRSP1* (Figure 3A). After the initial confirmation of *KK* in the index case (chrRCC), we screened a larger cohort and found *KLK4-KRSP1* isoform 1 (*KKv1*) expression restricted to 46 of 169 (27%) RCCs (Figure 3B). We also checked *KKv1* expression in matching normal kidney of the 46 cases and found that only ten had detectable levels of *KKv1*. Furthermore, our screen revealed that expression of the minor variant *KKv2* was restricted to only RCCs with highest *KKv1* expression (18 of 169 (11%)) (Figure 3C). Since *KKv3* was even weaker expressed than *KKv2* (see Additional file 1: Figure S2) and *KKv4* and *KKv5* were detected later as byproducts in a cloning effort to generate KK ORFs, the focus was set on screening expression of *KKv1* and *KKv2* only.

**Figure 4:**
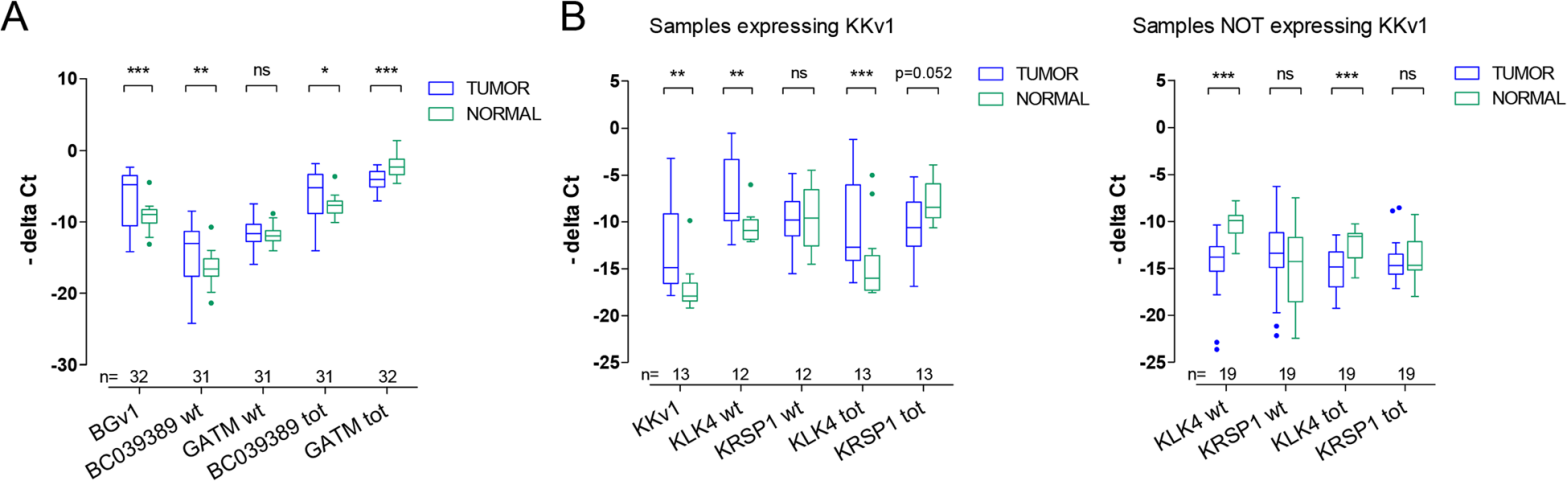
Expression levels of the read-throughs’ parent genes in tissues. (**A**) Expression of the *BC039389* and *GATM* parent genes alone (wt, wild-type levels) or with read-throughs (tot, total levels) in 32 frozen tumor and matched normal samples. One sample had not suffient RNA left for measuring wild-type and *BC039389* tot levels. The location of the TaqMan assays is indicated in Figure 2A. (**B**) Expression of the *KLK4* and *KRSP1* parent genes (wild-type and total transcript levels) in 13 samples expressing *KKv1* read-through (left panel) or 19 samples not expressing *KKv1* (right panel). To be consistent in showing normal tissues, we artificially calculated *KKv1* expression for 11 of 13 normal samples using the cut-off of Ct40, as *KKv1* was expressed only in 2 normal tissues of the *KKv1*-expressing cohort. The location of the TaqMan assays is indicated in Figure 3A. Significance was calculated using student’s *t*-test in paired samples (*p < 0.05, **p < 0.01, ***p < 0.001, ns = not significant).

We designed several TaqMan assays targeting the read-throughs’ parent genes to measure the expression profile of exons excluded (wild-type) or included (total) in the read-through transcripts (see Figures 2A and 3A for the location of the different assays). Tumors expressing *BGv1* had significantly elevated total (tot) and wild-type (wt) levels of the 5′ parent *BC039389* compared to matched normal kindey (Figure 4A). Despite elevated *BGv1*, total levels of the 3′ parent *GATM* were significantly reduced. *GATM* wild-type transcript was not differentially expressed. Similarly, tumors expressing *KKv1* also had significantly elevated total and wild-type levels of the 5′ parent *KLK4*, when compared to their matched normal tissues (Figure 4B, left panel). Similar to the reduction of *GATM*tot in *BGv1*-expressing samples, there was a trend of reduction seen for *KRSP1*tot in *KKv1*-expressing samples, too, although not significant. *KRSP1*wt remained unchanged. For the purpose of comparison, the measurements in tumors without any *KKv1* expression differed considerably (Figure 4B, right panel). There, *KLK4*tot and *KLK4*wt levels were significantly reduced when compared to their matched normal tissue. Taken together, this suggests that high expression of the 5′ parent might sway read-through expression and that a reduction of the 3′ parent may also contribute to read-through expression.

Having identified *BG* and *KK* as potential novel players in human RCC, we went on screening a panel of 47 cell lines. All cell lines expressed *BGv1* (Additional file 1: Figure S3A), 24 cell lines expressed *BGv2* (Additional file 1: Figure S3B) and *KKv1* expression was restricted to 13 cell lines (Additional file 1: Figure S3C).

### Clinical parameters of RCCs expressing *KLK4-KRSP1*

**Figure 5:**
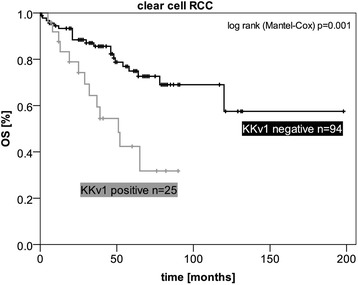
Survival analysis of *KKv1*-positive versus *KKv1*-negative ccRCCs. Kaplan-Meier curves evaluating overall survival (OS) of a cohort of ccRCCs with and without *KKv1* expression.

We performed some association analyses of *KKv1* expression with known clinical parameters. *KKv1* expression correlated significantly with larger tumors, high grade tumors and the histological subtype (Table 3). Notably, all chromophobe (chrRCC) and 50% papillary type 2 (pRCC type 2), compared to 20% clear cell (ccRCC) and 13% papillary type 1 RCCs in our cohort expressed *KKv1*. Survival analysis of the ccRCC sub-cohort (n = 119) showed a significantly reduced overall survival for patients with *KKv1* was expressing tumors (Figure 5).

**Table 3:** **Correlations of KKv1 expression with clinical parameters**

		**KKv1 expression**		**Spearman’s rho**
		**Yes**	**No**	
n		46/169 (27%)	123/169 (73%)	
age (y), median [range]		68 [32–86]	62 [22–86]	ns
tumor size (cm), median [range]		6.3 [2.1-17.5]	5.0 [1.2-18]	0.204 **
subtype	clear cell	25	102	0.274 **
	papillary type 2	7	7	
	papillary type 1	2	13	
	chromophobe	12	0	
	Xp11 translocation	0	1	
Fuhrman	G1-2	19	88	0.208 **
grade	G3-4	25	35	
	X	2	0	
pT	1-2	27	86	ns
	3-4	18	37	
	X	1	0	
N	yes	5	10	ns
	no	27	70	
	X	14	43	
M	yes	11	29	ns
	no	19	59	
	X	16	35	
sex	m	28	82	ns
	f	18	41	
VHL mutation	yes	7	50	ns
	no	3	15	
	unknown	36	58	

**Correlation is significant at the 0.01 level (2-tailed) in univariate analysis.

### *BC039389-GATM* and *KLK4-KRSP1* influence gene regulation in a converse manner compared to their respective parent transcript

**Figure 6:**
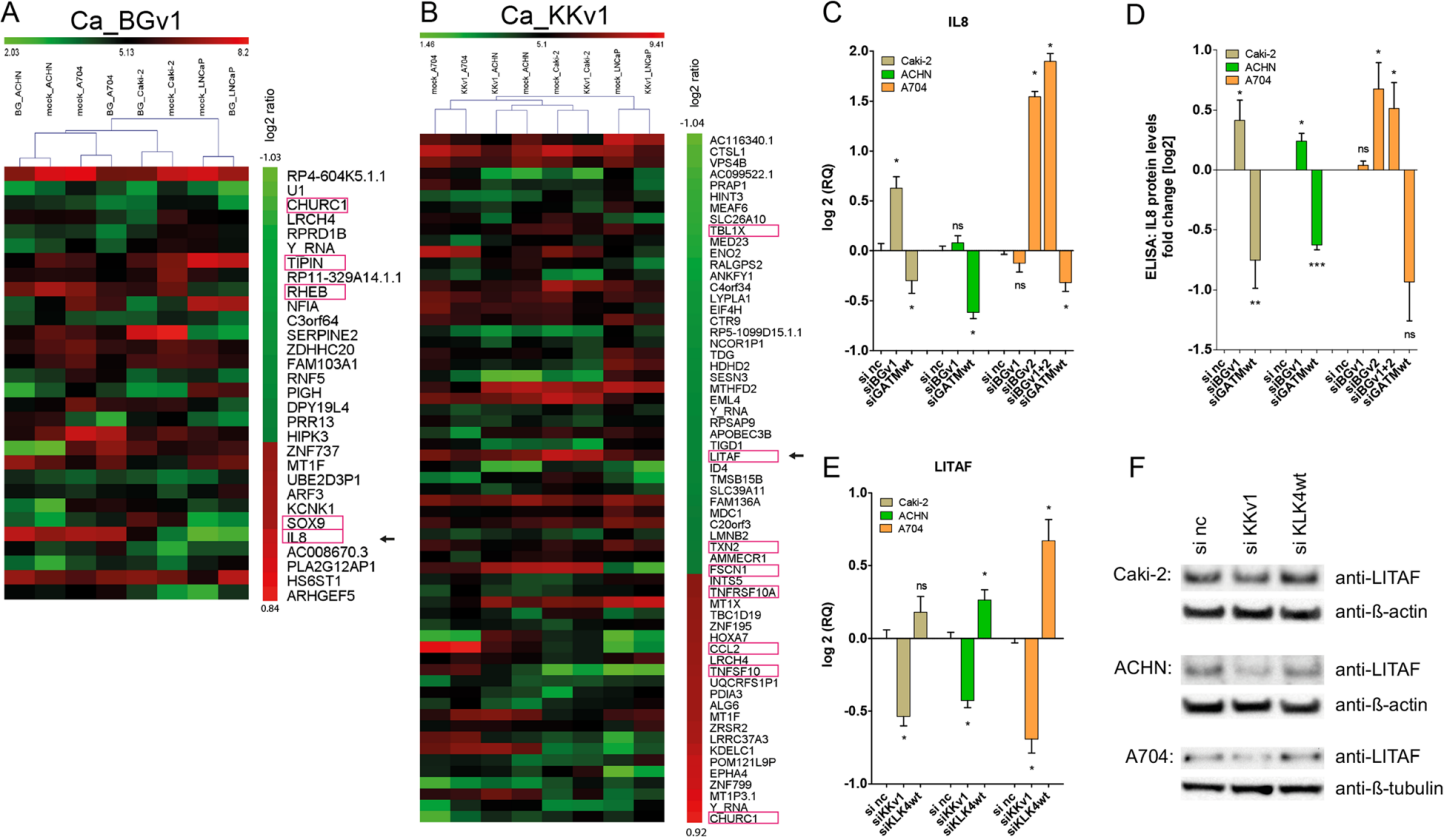
Functional consequences of *KLK4-KRSP1* and *BC039389-GATM* read-throughs on gene expression. (**A**, **B**) Heatmaps showing significantly dysregulated genes by *BGv1* (**A**) or *KKv1* (**B**) from genome-wide expression analysis of cancer (Ca) cell lines Caki-2, ACHN, A704, LNCaP, sorted from most down-regulated to most up-regulated in read-through knock-down vs. mock treatment. Pink rectangles mark the genes chosen for TaqMan qPCR validation of the data sets (see also Additional file 1: Figures S4 and S5). Arrows indicate the genes chosen for further experimentation. (**C**) *IL8* expression in *BG* and *GATMwt* knock-down cells compared to si negative control (si nc). (**D**) IL8 levels measured by ELISA in *BG* and *GATMwt* knock-down cells compared to si nc. (**E**) *LITAF* expression in *KKv1* and *KLK4wt* knock-down cells. (**F**) LITAF levels on Western Blot in *KKv1* and *KLK4wt* knock-down cells compared to si nc. Significance was calculated using student’s *t*-test (*p < 0.05, ns = not significant).

Having identified several cell lines expressing *BG* and *KK*, we were able to perform some functional analyses. We first searched for target genes by knocking-down the major isoforms of *BG* read-through (*BGv1*) and *KK* read-through (*KKv1*) in selected cell lines (Additional file 1: Figures S4A and S5A). Subsequent genome-wide microarray analyses revealed several significantly differentially regulated genes (Figure 6A,B & Additional file 1: Figures S4B-C and S5B-C) of which 13 with tumor-related functions (i.e. apoptosis, mTOR signaling, cell cycle regulation, directional cell movement) were selected for re-evaluation on RNA and protein level (Figure 6C-F & Additional file 1: Figures S4D-E & S5D). Most striking was the distinctively pronounced reciprocal regulation of *Interleukin 8* (*IL8*) in *BG* knock-down (si*BG*) versus the knock-down of the functional parent gene *GATM* (si*GATMwt*). si*BG* induced *IL8* whereas si*GATMwt* reduced it (Figure 6C). IL8 protein levels were too low to be detected by Western Blot. Therefore, we used ELISA to show that the *IL8* regulation was also apparent on protein level in the cell lines (Figure 6D). In concordance with the cell line data, we saw a trend of increased *IL8* levels in normal tissues (approximating the *BG* knock-down situation) and reduced *IL8* in tumor tissues (Additional file 1: Figure S6A).

A similar reciprocal regulation was observed for *Lipopolysaccharide-induced TNF factor* (*LITAF*) in *KKv1* knock-down (si*KKv1*) versus the knock-down of the functional parent gene *KLK4* (si*KLK4wt*). si*KKv1* reduced *LITAF* whereas si*KLK4wt* induced its expression (Figure 6E). LITAF protein was clearly detectable by Western Blot; but although there was a downregulation of LITAF upon *KKv1* knock-down, an upregulation of LITAF upon si*KLK4wt* was visible only weakly in Caki-2 and A704 (Figure 6 F). We concluded that Western Blot may not be sufficiently sensitive to detect the small increase in *LITAF* in all cell lines reliably. To substantiate the cell line data, quantitative measurements in tumor tissues were conducted and confirmed significantly reduced *LITAF* levels in RCCs without *KKv1* expression (*KKv1* negative, approximating the *KKv1* knock-down situation) compared to RCCs expressing *KKv1* (*KKv1* positive) having increased *LITAF* expression (Additional file 1: Figure S6B).

### *BC039389-GATM* and *KLK4-KRSP1* influence migration and invasion inversely compared to their respective parent transcript

**Figure 7:**
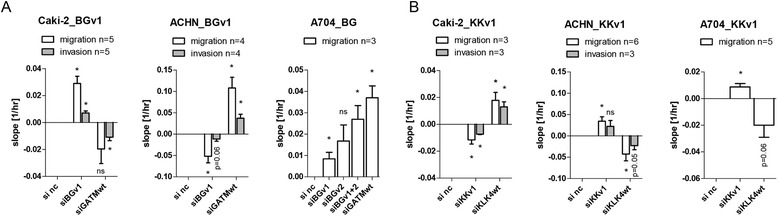
Functional consequences of *KLK4-KRSP1* and *BC039389-GATM* read-throughs on migration and invasion. (**A**, **B**) Invasion and migration changes in cell lines under read-through or parent gene knock-down in comparison to si negative control (si nc) (*p < 0.05 by student’s *t*-test). The migration/invasion of cells was measured in real-time and first plotted as delta cell index (no. of migrated cells) per given time period (see Additional file 1: Figure S7). Then the slopes of the curves of cells treated with targeting siRNAs were calculated in comparison to the baseline si nc curve and plotted as bar chart, where a minus slope indicates less migration, and a positive slope more migration than si nc, respectively. These experiments were performed as technical duplicates in biological replicates as indicated.

We observed such inverse effects of read-through and parent gene also on migratory and invasive properties of cell lines (Figure 7 & Additional file 1: Figure S7). ACHN had reduced migration/invasion, whereas Caki-2 had increased migration/invasion upon *BGv1* knock-down (Figure 7A). *GATMwt* knock-down had an opposing effect on ACHN (increase) and Caki-2 (decrease). *BG* knock-down with si*BGv1* or si*BGv2* alone or as double knock-down in A704 was performed although this cell line is minimally migratory and not invasive. All three *BG* knock-downs caused increased migration, but si*GATMwt* did not reverse this effect. *BGv1* knock-down had no gross influence on neither proliferation nor metabolic rates in cancer cell lines (Additional file 1: Figure S8). A small significant reduction in proliferation was seen upon *BGv1* knock-down in the normal kidney cell line HK-2.

We observed alterations of migratory and invasive capacities of cell lines under *KKv1* knock-down. ACHN and A704 had increased migration/invasion, whereas Caki-2 had reduced migration/invasion (Figure 7B). The knock-down with si*KLK4wt* could reverse the effect of si*KKv1* in all three cell lines. None of the cancer cell lines altered proliferative or metabolic rates under *KKv1* depletion (Additional file 1: Figure S8).

### *BC039389-GATM* and *KLK4-KRSP1* read-throughs likely belong to the non-coding RNA class

Despite all our efforts, we were unable to identify endogenous protein translated from *BG* or *KK* (see Additional file 1 and Additional file 1: Figures S9 and S10 for detailed experimentation). Although these experiments do not fully eliminate the possibility that the read-throughs still produce functional proteins, it suggests that the effects which we observed upon read-through knock-down are caused by the RNA molecules themselves. We found *KKv1* enriched in the nuclear fraction of RCC cells (Additional file 1: Figure S11A), possibly indicating a role in transcription regulation. *BGv1* was more abundantly expressed in the cytosol (Additional file 1: Figure S11B), possibly suggesting a role in processes taking place in this cellular compartment. Further experiments are mandatory to pinpoint the mechanism(s) by which the read-throughs are exerting their function.

## Discussion

This is the first study that aims at elucidating the role of read-through RNA chimeras in RCC. We identified read-throughs *B039389-GATM* and *KLK4-KRSP1* up-regulated in RCC compared to normal kidney. We show that these read-throughs are able to functionally oppose at least one of their two parent genes exemplified by altering migration and invasion properties of cell lines and by inversely regulating *IL8* (*BG*) and *LITAF* (*KKv1*).

A direct comparison of our 324 read-through calls obtained from RNA-Seq in RCC with six in breast cancer [8] and 339 in prostate cancer [9] revealed only a marginal overlap of identical read-through calls. Such discrepancies may be explained by diverging expression of RNA read-throughs in different cancer types, but also by the non-uniformity of the employed data filtering strategies.

Only a few reports describe a putative functional impact of read-throughs on cancer [4,8] with the most convincing one reported for *SLC45A3-ELK4* in prostate cancer, where the read-through promotes cell proliferation but wild-type *ELK4* does not [4]. Our study is the first one in which functional consequences of read-throughs were investigated in RCC.

The function of *BC039389*, one of the parent genes of *BG*, is yet unknown. The second parent gene *GATM* catalyzes the rate limiting step of creatine synthesis and its expression is seen in various tissues, with the highest levels observed in kidney. It was shown that both the protein (Additional file 1: Figure S9C and [18]) and the mRNA (Oncomine database) expression of GATM is reduced in RCC compared to normal kidney. We hypothesize that after transcription of *GATM,* the 3′ exons (i.e. exon 7, *GATM*tot) are more susceptible to degradation compared to the 5′ exons (i.e. exon 1, *GATM*wt). This may explain both the reduction of *GATM*tot in RCC and the unchanged expression of G*ATM*wt when compared to normal kidney, seen in our study. Steady transcription of exon 1 is probably required to be spliced for the sake of generating *BG*. Even under the condition of reduced *GATM* in tumor, there still exists a manifold excess of *GATM* mRNA over *BG* read through, preventing us from detecting the increase of *BG* when measuring *GATM*tot in tumor. We suspect that *BG* expression is not only driven by *BC039389* increase, but that also *GATM* loss plays a role since such loss evokes an increase of *BG* expression, as reflected by the significant induction of *BGv1* by 1.5- to 2-fold in the experiment with si*GATMwt* knock-down in ACHN and Caki-2 (Additional file 1: Figure S7A). We used microarrays to find out whether *BGv1* influences the expression of genes. The most prominent target gene was *IL8*, a cytokine. IL8 regulation is critical in a lung metastasis mouse model where it was shown that RCC cells with high IL8 have less competence to metastasize because they seem to attract tumor-cytotoxic neutrophils to the metastatic niche in the lung [19]. Preventing the recruitment of such killer neutrophils through downregulation of IL8 may well be one of the synergistic functions of reducing GATM and increasing *BG* in cancer cells. Hence, we suspect a rather tumor-promotive function of *BG*. We can observe this in form of reduced migration/invasion of ACHN cells upon *BGv1* knock-down, but this effect is cell line-dependent. Regarding the effects on IL8 and migration/invasion as two independent examples of reciprocal regulation, we can corroborate the theory that the read-through transcript *BG* and wild-type GATM are functionally opposing each other in such a manner that ultimately the result may be beneficial for tumorigenesis.

Recently, the *KLK4-KRSP1* read-through has been described in a publication on pseudogenes [20]. The authors describe high expression of, what we named, *KKv1* in prostate cancer. Both parent genes of this read-through are part of the large family of tissue kallikreins. With the emerging role of tissue kallikreins in cancer [21], KLK4 expression has been profiled in several cancer tissues e.g. prostate [22], ovary [23], endometrium [24] and colon [25]. Elevated KLK4 levels were frequently associated with higher tumor grade in endometrial and prostate cancer [24,26] and poor prognosis in ovarian cancer [23]. Kallikreins are secreted proteases and KLK4 appears to be the only representative of this family with pro-proliferative function mainly localized to the nucleus [22]. Little is known about the function of *KRSP1*. It potentially can be translated into a protein which is primarly present in the nucleus [27]. Also for *KK*, we hypothesize that the 5′ parent’s expression is driving the read-through’s expression, as *KLK4*tot and *KLK4*wt levels are higher in tumors expressing *KKv1* than in their matched normal kidney tissues. Similar to our hypothesis of an interplay between *GATM* loss and *BG* increase, the parent genes’ expression data for *KK* is hinting towards a role of *KRSP1* loss in *KK* expression. We were unable to directly proof such a link, since we could not design a siRNA targeting specifically *KRSP1*wt (as pseudogene, *KRSP1* (exons 1–3) has great homology to all members of the family of tissue kallikreins) to perform a *KRSP1*wt knock-down eventually increasing *KKv1* expression. After microarray analysis, we found *LITAF* as a major target gene of *KKv1*. The literature about *LITAF* function in cancer is inconsistent. One report mechanistically proofs *LITAF* as p53-repressed [28]. Putting this in the context of RCC, where loss of pVHL in 70% of ccRCCs may contribute to destabilization of p53 [29], one could expect increased *LITAF* in these ccRCCs. With *KKv1*, we have found yet another player in *LITAF* up-regulation expressed in 20% of ccRCCs. We also report *KKv1* expression frequently associated with pRCC type 2 and chrRCC, histological RCC subtypes in which VHL and p53 are largely functional. There, *LITAF* up-regulation could be mediated almost exclusively by *KKv1*. Since we observe worse survival for patients in the presence of *KKv1*, we would attribute *KKv1* rather tumor-promotive properties. However, a tumor-promotive effect of *KKv1* on migration/invasion of RCC cells is cell line-dependent and may be one reason why *KKv1* did not correlate with local or distant metastasis in patients.

Although previous reports suggest that some read-throughs encode fusion proteins [5,8,16,17], we and others [7] suspect that most read-throughs suit the class of non-coding RNA. In accordance with Prakash et al. [7], most of the read-throughs we are reporting here are not predicted to encode fusion proteins. Any analysis of endogenous protein levels is difficult in consideration of the low levels of read-through expression. Low RESPER scores and high Ct values (Ct 27–40 compared to Ct 18 for the reference gene PPIA) demonstrate that read-throughs are mostly transcribed at rather low rate. And despite all our efforts, we have found no proof of protein expression from either *BG* or *KKv1*.

In summary, we demonstrate for the first time that read-throughs act as regulators by counteracting their parent genes in suppressing or activating genes or mechanisms in renal cancer. Yet, how they regulate and how they themselves are regulated is still to be determined. Most of our read-throughs were similarly expressed in tumor and normal tissue. However, the fact that *BC039389-GATM* and *KLK4-KRSP1* are two examples of read-throughs expressed at higher levels in tumor tissue hints towards a rather oncogenic function for some read-throughs. This coincides with the hypothesis that RNA chimerism is more frequently observed in prostate cancer than in benign prostate tissue [11].

## Conclusions

A growing body of literature on RNA chimeras indicates that chimeric read-through transcripts have implications in cancer. We show that such read-through transcripts are abundantly expressed in renal cell carcinoma and, on the basis of two examples *BC039389-GATM* and *KLK4-KRSP1*, may be tumor-promoting and impacting cellular properties in a way which inverses the effect of their wild-type parent transcripts.

## Methods

### RNA extraction

RNA extractions from fresh frozen RCC tissues, matched adjacent normal kidney tissue and cell lines were performed with RNeasy kit (Qiagen). Prior to ethanol precipitation the frozen tissues were cut to 10 μm thick sections and homogenized in RTL buffer + β-Mercapthoethanol for 2 min at 30Hz (twice) in a TissueLyser (Qiagen). Cell lines were homogenized using QIAshredder columns (Qiagen). Our retrospective study fulfilled the legal conditions according to Article 34 of the Swiss Law “Humanforschungsgesetz (HFG)”, which, in exceptional cases, allows the use of biomaterial and patient data for research purposes without informed consent, if i) it is impossible or disproportionately difficult to obtain patient consent; ii) there is no documented refusal; iii) research interests prevail the individual interest of a patient. Law abidance of this study was reviewed and approved by the ethics commission of the Canton Zurich (KEK-ZH-Nr. 2011-0072/4).

### Whole transcriptome sequencing and computational read-through analysis

Seven fresh frozen cancer tissues were subjected to paired-end (PE) whole transcriptome sequencing on Illumina GAII followed by nomination of chimeric transcripts by the analysis software FusionSeq as previously described [30]. Only read-through candidates, characterized by PE reads covering two neighboring genes on the same DNA strand, were considered for this study. Inter-, intra- and cis chromosomal candidates were disregarded.

### Candidate validation by RT-PCR and quantitative PCR (qPCR)

Frozen RCC and normal kidney tissue was used for candidate validation. The putative RNA junction region of selected read-throughs, narrowed down by FusionSeq, was amplified by at least two different primer combinations (Additional file 2: Table S1). Resulting amplicons were separated on agarose gels, extracted and Sanger sequenced to proof their identity (Additional file 1: Figure S2). Quantitative TaqMan assay design was based on the results gained from Sanger sequencing and allowed reliable detection mostly for the prominent isoform(s) of a given read-through event as indicated in Table 1. Primers and probes were purchased from Microsynth AG (Balgach, CH). TaqMan qPCRs were performed on Real-Time PCR Systems from Applied Biosystems using the TaqMan RNA-to-Ct 1-step kit (Life technologies) in the presence of 0.2 μM of each primer and 0.9 μM of the probe. Differential expression of all genes was calculated relative to the endogenous control *PPIA*.

### Cell culture

Cell lines were purchased from ATCC (Molsheim Cedex, F) and cultivated accordingly. The benign kidney cell line HK-2 was propagated in medium K-1 which was originally described [31] and consists of a 1:1 mixture of DMEM and Ham’s F-12 Nutrient mixture supplemented with a mixture of hormones (2.5 μg/ml insulin, 0.625 ng/ml prostaglandin E_1_, 16.9 pg/ml (or 26 pM) 3,3,5 Triiodothyronine T3, 2.5 μg/ml transferrin, 9 ng/ml (or 25 nM) hydrocortisone). Additionally, we supplemented with 5% FCS, 1% penicillin/streptomycin, 25 mM HEPES, 5 ng/ml EGF and 0.865 ng/ml (or 50pM) sodium selenite. All cell lines were authenticated (Microsynth).

### siRNA treatment

Read-through specific knock-down was achieved by placing the siRNA into the exon junction of *KKv1* and *BGv1* with at least 8 nucleotides covering each parent gene. Read-through specific-, *Lamin A/C* (*LMNA*, pos. ctrl.) and non-targeting (si nt = si non-targeting) siRNAs were purchased from Microsynth AG. For sequences see Additional file 3: Table S2. AllStars Negative Control siRNA (si nc) was purchased from Qiagen. Cell line-specific knock down protocols were established using siRNA concentrations of 24nM, 48nM, 72nM, 120nM and 240nM. Chosen was the concentration where *LMNA* pos. ctrl. was efficiently knocked-down (>50%) and nt siRNAs had no effect. Time points for testing knock-down effect were 24 h, 48 h, 72 h and 96 h. Chosen was the time point when read-through specific siRNAs had sufficient knock-down (>70%) but wild-type parent genes were unaffected (see Additional file 1: Figures S4, S5, S7 and S8). Optimal transfection reagents for each cell line were titrated using pos. ctrl. siRNA: Caki-2, ACHN, LNCaP and HK-2 were pre-seeded over-night before transfection with HiPerFect (Qiagen), A704 was pre-seeded over-night before transfection with Lipofectamine RNAiMAX (Life technologies) and HEK293 were transfected using a fast-forward protocol with Lipofectamine 2000. Both lipofectamine agents required a medium change after 6 h (A704) or 10 h (HEK293).

### Microarray gene expression analysis of read-through-specific knock-down cell lines

For genome-wide expression analysis, only RNA samples with >85% *KKv1* or *BG* knock-down were used (Additional file 1: Figures S4A and S5A). RNA quality control, sample preparation, chip run (human Affymetrix Gene 1.1 ST Array strips) and data pre-processing were performed by the Functional Genomics Center Zurich (FGCZ). Data analysis was done using B-Fabric, an open software provided by the FGCZ. Only genes with a log2 ratio ≥ (±)0.5 and an uncorrected p-value ≤0.05 between mock (transfection reagent) and siRNA treated cell lines were considered. Heatmaps were constructed using the MultiExperiment Viewer (MeV). Regulation of selected target genes, relative to *PPIA*, was evaluated by TaqMan qPCR (Additional file 1: Figures S4D and S5D).

### ELISA

To measure IL8 protein levels by ELISA, siRNA knock-downs were performed in cell lines Caki-2 (60 000 cells/well of a 12-well plate) and ACHN (150 000 cells/well of a 12-well plate) for 48 h, and A704 (125 000 cells/well of a 12-well plate) for 72 h. Cells were harvested by trypsinization, the pellet was washed with PBS and the whole cell lysate was extracted using 30ul of RIPA buffer. Protein concentration was measured by Pierce BCA Protein Assay Kit (Thermo Scientific). Twice 10ul of the whole cell lysate was used in the ELISA assay (Human IL8 ELISA Ready-SET-Go! (2^nd^ Generation) (eBioscience Affymetrix, Austria)). The subtracted absorbance of the samples’ duplicates was compared to the standard curve using the 4-parameter logistic curve fit in GraphPad Prism. The resulting mean IL8 concentrations of the samples were normalized to the protein content in the whole cell lysate. Then, the fold change between the respective si nc samples and knock-downs of the read-throughs or wild-type genes was calculated and plotted as mean + SEM for technical quadruplicates.

### Migration/invasion assays

Migratory/invasive potential of RCC cell lines was measured in real-time using the xCELLigence RTCA DP System (ACEA Biosciences, San Diego). This technique adapts the boyden chamber principle and combines it with impedance measurements. Cells seeded in the upper chamber of a microplate containing “low chemoattractant (1% FCS)” can migrate through the microporous membrane into the lower chamber being the “high chemoattractant (10% FCS)” compartement. Migrated cells adhere to the gold micro-electrode sensor located at the lower side of the membrane and lead to an increase in impedance, which is measured by the RTCA DP instrument. Knock-down of read-throughs, wild-type parent genes and negative control siRNA were performed for 48 h (BG: ACHN,Caki-2; KK: A704) and 72 h (BG: A704; KK: ACHN, Caki-2) (Additional file 1: Figure S7A) before seeding the cells into the microplates in duplicates. Optimal cell seeding densities were determined in pre-experiments. Impedance measurements were performed for 72 h (Additional file 1: Figure S7B-C). For invasion assays, the membrane was coated with Matrigel Basement Membrane Matrix (BD Biosciences, 400ug/ml protein in 1% FCS-containing medium).

### Availability of supporting data

The junction sequences of the read-throughs are deposited to Genbank under accession number [GenBank:KM576708-KM576757].

## Additional files

Additional file 1:
**Describes in detail** **(i)** **the results of the experiments performed to substantiate or negate protein translation from the read-throughs,** **(ii)** **additional Material and Methods omitted from the main manuscript because they were used in experiments featured only in additional figures and** **(iii)** **additional figures 1–11 with figure legends.**
Additional file 2: Table S1. Lists the primer and probe sequences. Additional file 3: Table S2. Lists the siRNA sequences.

## Abbreviations

BG: BC039389-GATM read-through
BGv1: BC039389-GATM transcript variant 1
BGv2: BC039389-GATM transcript variant 2
ccRCC: Clear cell Renal Cell Carcinoma
chrRCC: Chromophobe Renal Cell Carcinoma
FCS: Fetal calf serum
HA: Hemagglutinin
KK: KLK4-KRSP1 read-through
KKv1: KLK4-KRSP1 transcript variant 1
KKv2: KLK4-KRSP1 transcript variant 2
nc: negative control
ns: not significant
nt: not targeting
pRCC: papillary Renal Cell Carcinoma
qPCR: quantitative PCR
RCC: Renal Cell Carcinoma
RESPER: Ratio of empirically computed supportive paired-end reads
RT-PCR: reverse transcription-PCR
tRCC: Xp11 translocation RCC
