# Supplementary material for: Functional characterization of BC039389-GATM and KLK4-KRSP1 chimeric read-through transcripts which are up-regulated in renal cell cancer
Source: BMC Genomics. 2015 Mar 27;16(1):247. doi: 10.1186/s12864-015-1446-z (PMC4422297; doi:10.1186/s12864-015-1446-z)
Supplement: Additional file 1: — Describes in detail (i) the results of the experiments performed to substantiate or negate protein translation from the read-throughs, (ii) additional Material and Methods omitted from the main manuscript because they were used in experiments featured only in additional figures and (iii) additional figures 1–11 with figure legends. [file 12864_2015_1446_MOESM1_ESM.docx]

**Additional file 1**

***BC039389-GATM* and *KLK4-KRSP1* read-through transcripts are up-regulated in renal cancer and influence cellular mechanisms in an opposing manner compared to their parent transcripts *GATM* and *KLK4***

Dorothee Pflueger^1,2^, Christiane Mittmann^1^, Silvia Dehler^3^, Mark A. Rubin^4,5^, Holger Moch^1,2,*^, Peter Schraml^1,*^

^1^ Institute of Surgical Pathology, University Hospital Zurich, Zurich, Switzerland

^2^ Competence Center Personalized Medicine, ETH and University of Zurich, Switzerland

^3^ Cancer Registry Cantons Zurich and Zug, University Hospital Zurich, Zurich, Switzerland

^4^ Institute of Precision Medicine of Weill Cornell Medical College and New York-Presbyterian Hospital, New York, USA

^5^ Department of Pathology and Laboratory Medicine, Weill Cornell Medical College, New York, USA

^*^ shared senior authorship

**Supplementary Results**

***BC039389-GATM* and *KLK4-KRSP1* are likely not translated into protein**

Using an alternative start codon downstream of the native ATG, both *BG* isoforms encode a N-teminally truncated GATM (**Supplementary Figure 8A**). Using anti-GATM antibody, we detected protein bands of predicted size for GATM and BG on Western Blots of cell lines expressing *BG* (**Supplementary Figure 8B**). The putative BG protein band was especially strong in RCC and benign kidney cell lines. In a Western Blot of matched tumor/normal human samples, GATM was reduced in tumor samples, as expected (**Supplementary Figure 8C**). However, the putative BG band was stronger in normal tissue, contrary to our expectation. To further investigate if the protein band around 35kDa was BG, we performed immunoprecipitation (IP) to enrich the putative BG followed by mass spectometry, but were not able to find GATM peptide sequences (**Supplementary Figure 8D-F**). As positive controls for the Western Blot, we loaded lysates from HEK293T cells exogenously over-expressing GATMwt and BG. Moreover, we performed *BG* knock-down in cell lines that endogenously express *BG* (ACHN & HK-2), but were not able to detect a change of BG band signal, not even when we included an siRNA targeting *GATM* exon 7, representing *BG* as well as wild-type *GATM* transcripts (**Supplementary Figure 8G-H**). The knock-down of *GATMwt* was indeed visible. We concluded that the anti-GATM antibody is reliably detecting GATMwt but had unspecifically detected another endogenous protein which unfortunately has the predicted size of BG.

*KLK4-KRSP1* produces at least 5 isoforms all of which potentially encode protein with more (KKv2, KKv2alt) or less (KKv1, KKv1alt, KKv3, KKv4, KKv5) similarity to wild-type KLK4 (**Supplementary Figure 9A**). Both, *KKv1* and *KKv2* RNA isoforms were found to include normal exon 2 or an alternatively spliced version that lacks the first 19 bases of this exon. The splicing event produces a frameshift causing a subsequent STOP codon (fsX6) and allows the use of a downstream located alternative start codon (KKv1alt, KKv2alt). The same alternative start codon is used for KLK4 in the LNCaP PCa cell line [[1](#_ENREF_1)]. To investigate a possible translation from *KK* transcript into protein, we cloned the putative KKv1, KKv2 and KLK4wt ORFs from the native and the alternative ATG (alt) to express them in HEK293T and ACHN cells (**Supplementary Figure 9B-C**). Knowing that *KLK4* is an androgen-regulated gene, we tried to upregulate endogenous KK expression in the androgen-dependent PCa cell line LNCaP and in the RCC cell line Caki-2. Although *KK* RNA levels were increased with DHT (dihydrotestosterone) in LNCaP and with MG132 (proteasomal inhibitor) in Caki-2 (**Supplementary Figure 9D-E**), we could not induce any KK protein production (**Supplementary Figure 9F-G**). Since KKv2 and KLK4wt are both proteins of same size, one cannot reliably distinguish between the two at Western Blot level. When we tried to precipitate endogenous KKv1 by IP from UOK146 (a Xp11 translocation RCC cell line with high levels of *KKv1* (**Supplementary Figure 9H**)), we could not detect a band of same size as the reference KKv1 protein, although the antibody could precipitate KKv1 and KLK4wt from the HEK293T positive controls (**Supplementary Figure 9I**). Only a band corresponding to KLK4wt was precipitated.

**Supplementary Material and Methods**

**Cell proliferation and viability/metabolism assays**

Cell proliferation and metabolic rates upon read-through-specific knock down were measured using the Cell proliferation ELISA, BrdU (colorimetric) and Cell proliferation Kit I (MTT) kits from Roche. After siRNA treatment of cells with indicated amounts and time points, cells were trypsinized, counted and seeded in 96-well formats. After 3-6h time to adhere, BrdU was added for 19-24h and further processing was performed according to the manufacturer’s manual with adding the stop solution. After 19-24h time to adhere, the MTT assay was performed according to the manufacturer’s instructions. Cell count after siRNA treatment was determined by the NucleoCounter NC-100 (Chemometec, Allerod, DK). BrdU and MTT absorbance was measured by a Tecan Infinite 200 Pro Tecan reader.

**Hormone stimulation experiments**

Cell lines were deprived of hormones for 48 h by seeding them at 70% confluency in phenol-red free medium supplemented with 5% charcoal-stripped FCS. Dihydrotestosterone (DHT) (5α-Androstan-17β-ol-3-one, Sigma), MG132 (protease inhibitor, Sigma) or mock (100% ethanol or DMSO, respectively) were added for 12h (LNCaP) or 18h (Caki-2) before cells were harvested for RNA extraction. Optimal concentrations of DHT and MG132 were determined in pre-experiments.

**Plasmid construction and transfection of BG and KK ORFs**

The open reading frames (ORFs) of *KKv1, KKv2, BG* and the coding parent genes were extracted from two tumor RNA samples (see **Supplementary Table 1** for primer sequences). The ORFs were tagged with one HA-sequence at the C-terminus, cloned into the pcDNA3.1(+) vector (Invitrogen) using BamH1 and EcoR1 restriction sites and propagated in XL1-Blue chemically competent bacteria (Stratagene). HEK293T cells were transfected using X-tremeGENE HP (Roche). After test experiments with a GFP-expressing vector, >80% transfection efficiency was reached with 2µl transfection reagent and 0.7µg plasmid/100 000 cells. ACHN cells were transfected using Attractene (Qiagen) (optimal efficiency with 1.5ul transfection reagent and 0.5ug plasmid/100 000 cells). For Attractene, a medium exchange after 6h was necessary.

**Immunoprecipitation of BG and KK ORFs**

Immunoprecipitation was done using the Pierce Classic IP Kit (Thermo Scientific). The cell lysis was done in a Tris non-denaturing lysis buffer (150mM NaCl, 1% Triton X-100, 1mM EDTA, 50mM Tris, 2mM NaF, 2mM sodium orthovanadate (Na3VO4)). 5mg (ACHN, A704, UOK146) or 5-50ug (KK ORF over-expressing HEK293T) whole cell protein lysate was pre-cleared with respective amounts of control agarose resin for 1h. The IP antibody (10ug anti-GATM for ACHN and A704, 10ug anti-KLK4 for UOK146, 1ug anti-KLK4 for HEK293T) was added to the lysate and incubated over-night. Then, the incubation with protein A/G was done for 2h. Washing of the protein A/G-captured antibody/lysate mixture was performed using the Tris non-denaturing lysis buffer and repeated 8-10 times. Elution was done with the low-pH elution buffer. The sample buffer elution protocol was disregarded as it produced a distinct undesirable band pattern on Western Blot. The precipitated protein was mixed with NuPAGE LDS sample buffer (Life technologies) and loaded on a 10% Bis-Tris gel (NuPAGE) in the presence of MOPS running buffer to optimize the low molecular weight protein separation. The bands were made visible with HRP-conjugated secondary anti-rabbit antibody.

**Antibodies for Western Blot**

anti-GATM (GeneTex, GTX114423), anti-KLK4 (Abcam, ab28564), anti-MCL-1 (cell signaling, 5453), anti-CANT1 (abnova, H00124583-M01), anti-LITAF (Abcam, ab88742), anti-actin (Chemicon, MAB1501), secondary anti-mouse IgG (Thermo Scientific, 31430) and secondary anti-rabbit IgG (Thermo Scientific, 31460).

**Cellular fractionation**

The fractionation of cellular compartments of Caki-2, ACHN and A704 cell lines was conducted according to the protocol detailed in Zaghlool A. *et al.* [[2](#_ENREF_2)] using the Cytoplasmic & Nuclear RNA purification kit (Norgen Biotek Corp., Canada) with the following specifications:

(a) 3 Mio cells per cell line were trypsinized, washed with PBS and lyzed in 300ul cold Buffer J by pipetting up and down for 5 min on ice.

(b) 150ul of this lysate was taken (including the membraneous conglomerate), transferred to a new tube and used for extraction of cytoplasmic RNA.

(c) The remaining clean lysate was used to extract nuclear RNA.

(d) After centrifugation, the fractions were applied to a sucrose cushion where both phases consisted of 1.6M sucrose with RNase inhibitor, as outlined by Zaghlool *et al*.

(e) The nuclear lysate was passed several times through a 25 gauge needle according to recommendations.

(f) An on-column DNAse digest was conducted.

Quantitative measurement of *KKv1* and *BGv1* in the RNA extracted from the two compartments was done using the TaqMan RNA-to-Ct 1-step kit. Read-through expression was normalized to a reference because the total RNA amount used in the qPCR was much less in the nuclear fraction than in the cytoplasm. Therefore, the raw Ct values are not comparable. Due to the absence of a reference that is expressed equally in both compartments, we chose a housekeeping gene (*PPIA* mRNA) to approximate an enrichment in the one or the other compartment, similar to what Zaghlool *et al*. did in their data analysis. *PPIA* was still measurable in both compartments because a fractionation can only enrich the compartments, but never completely separate them. Normalized levels in the cytoplasm was the ground level (set to 0) to which the nuclear levels were compared to in each cell line.

**Supplementary Figures**

**Supplementary Figure 1:**

324 read-through candidates nominated by FusionSeq sorted by sample and plotted in descending order of RESPER scores.


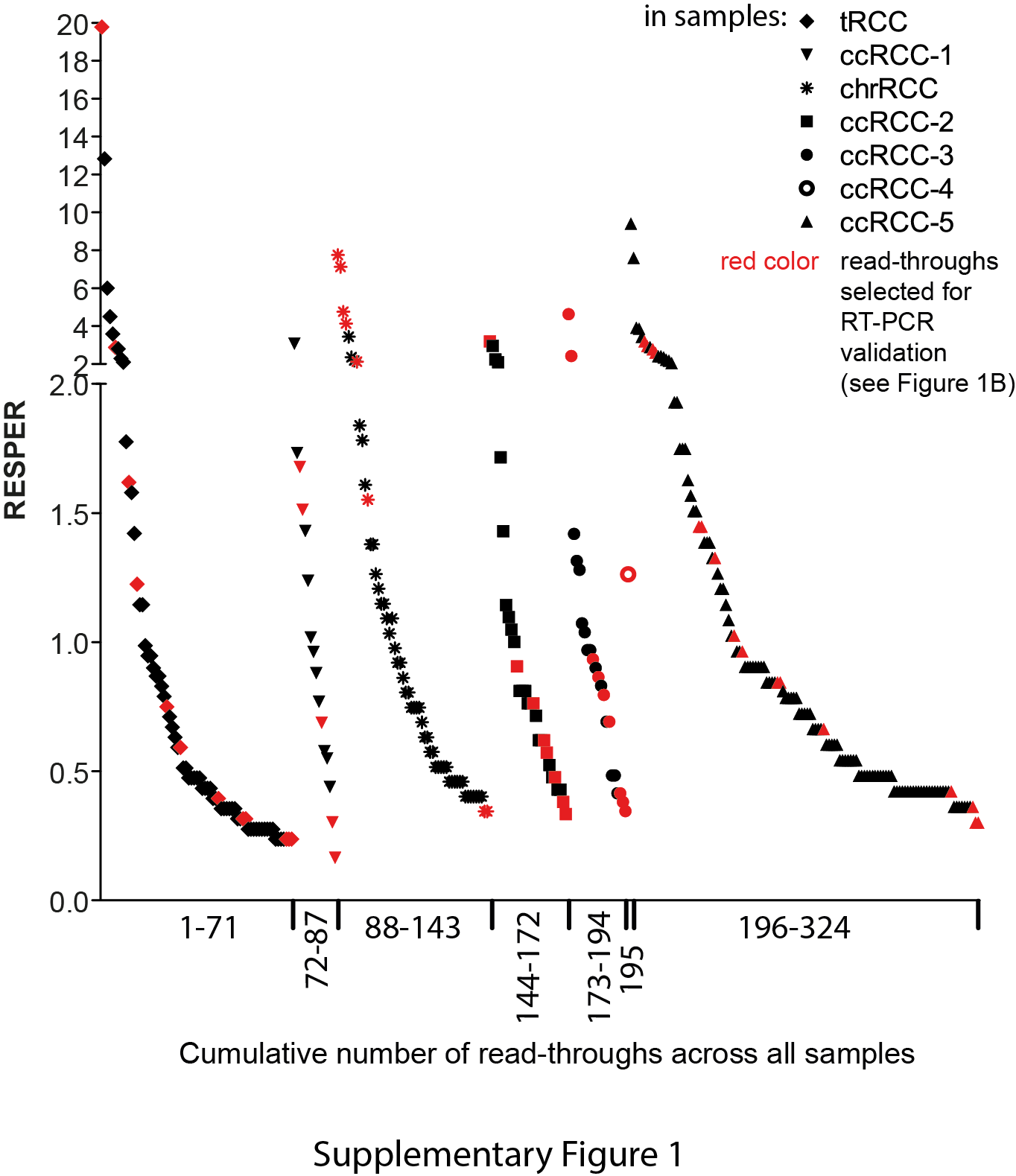


**Supplementary Figure 2:**

Experimental validation of 27 read-through candidates using RT-PCR. At least two distinct primer combinations verify or negate the expression of the read-through candidates.


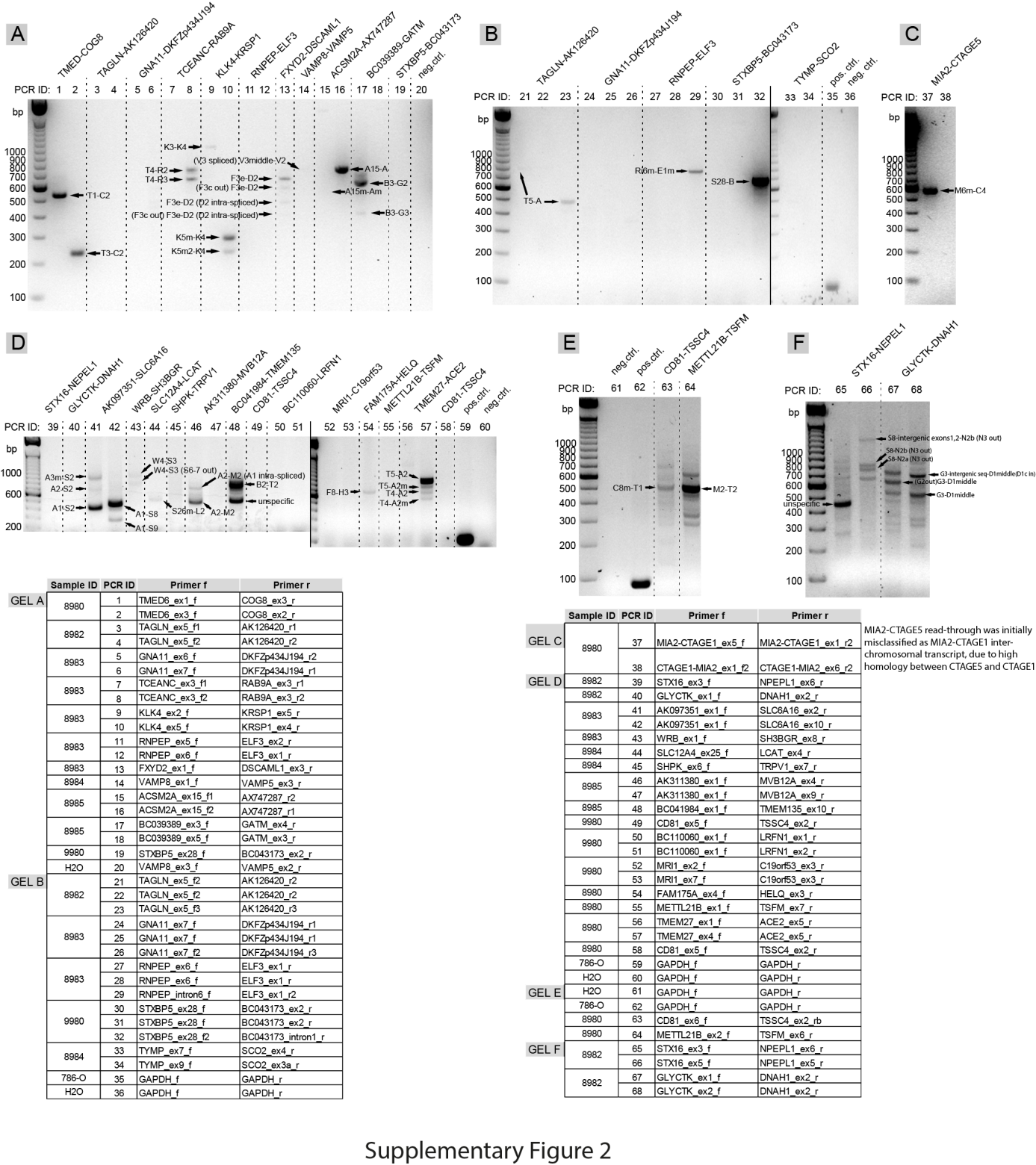


**Supplementary Figure 3:**

Expression of **(A)** *BGv1*, **(B)** *BGv2* and **(C)** *KKv1* in a panel of cell lines.


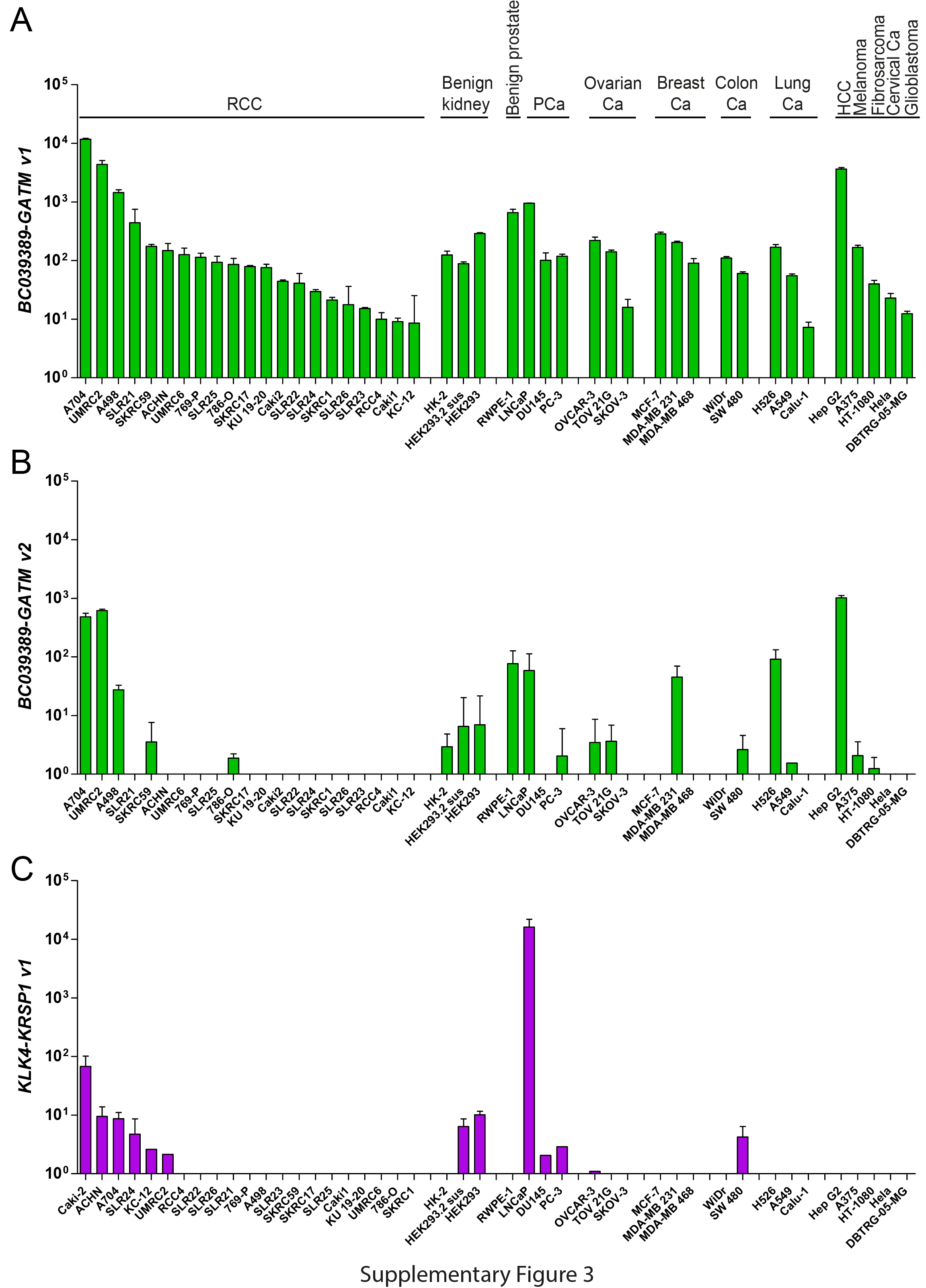


**Supplementary Figure 4:**

Genome-wide expression analysis in *BGv1* knock-down cells. **(A)** *BGv1* knock-down verification in 5 cell lines subjected to microarray expression analysis. **(B-C)** Heatmaps showing significantly dysregulated genes by *BGv1* from genome-wide expression analysis of kidney cancer cell lines Caki-2, ACHN, A704 (**B**) or all cell lines (**C**), sorted from most down-regulated to most up-regulated in read-through knock-down vs. mock treatment. **(D)** Target gene evaluation by TaqMan qPCR (log2 of relative expression/quantitation (RQ) compared to si negative control (si nc, si nt)) in *BG* and *GATMwt* knock-down cells (* p<0.05, ns=not significant by student’s t-test). **(E)** Inverse regulation of SOX9 on protein level by si*BGv1* and si*GATMwt* in ACHN.

**
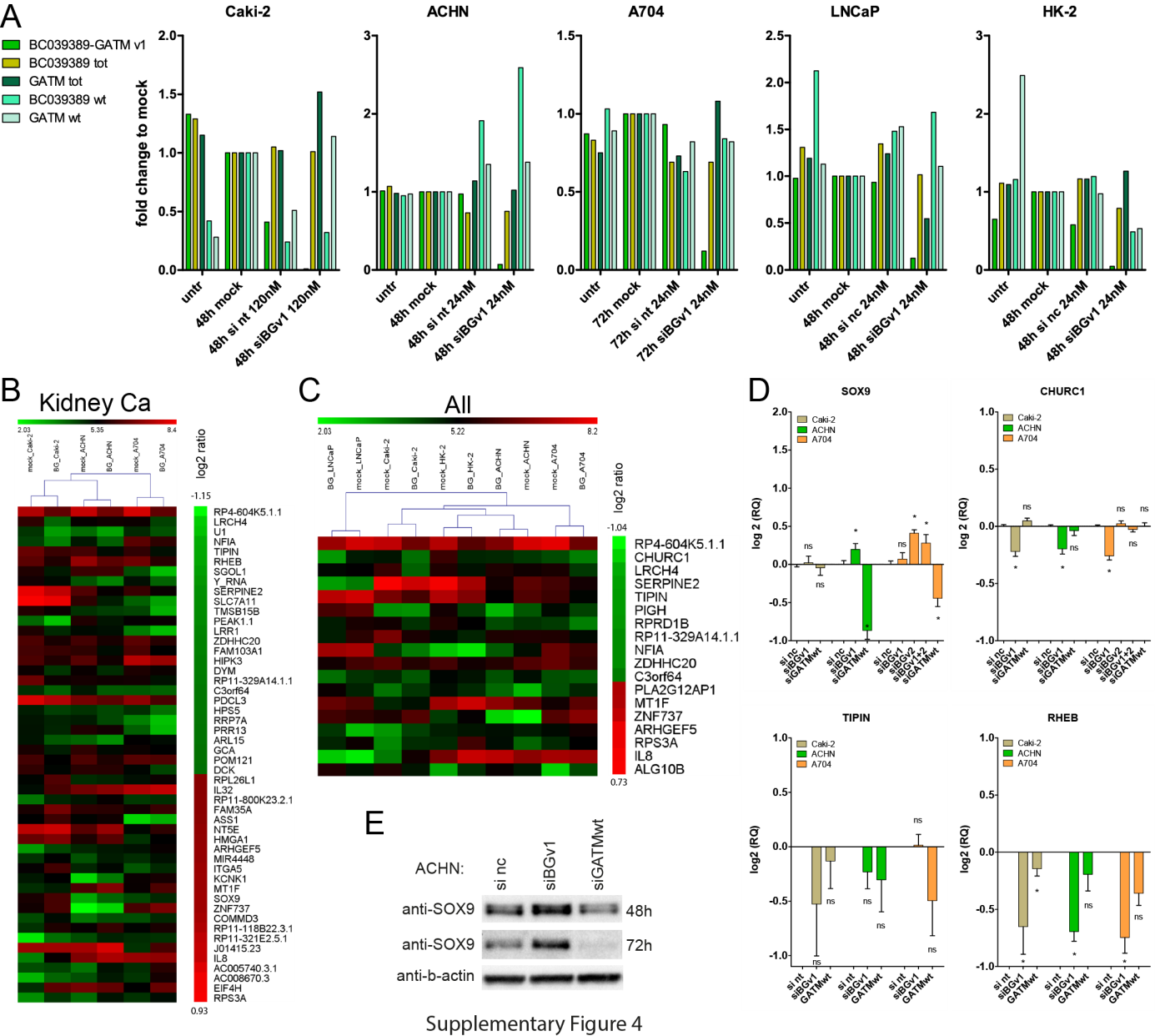
**

**Supplementary Figure 5:**

Genome-wide expression analysis in *KKv1* knock-down cells. **(A)** *KKv1* knock-down verification in 5 cell lines subjected to microarray expression analysis. **(B-C)** Heatmaps showing significantly dysregulated genes by *KKv1* from genome-wide expression analysis of kidney cancer cell lines Caki-2, ACHN, A704 (**B**) or all cell lines (**C**), sorted from most down-regulated to most up-regulated in read-through knock-down vs. mock treatment. **(D)** Target gene evaluation by TaqMan qPCR (log2 of relative expression/quantitation (RQ) compared to si negative control (si nc, si nt)) in *KKv1* and *KLK4wt* knock-down cells (* p<0.05, ns=not significant by student’s t-test).

**
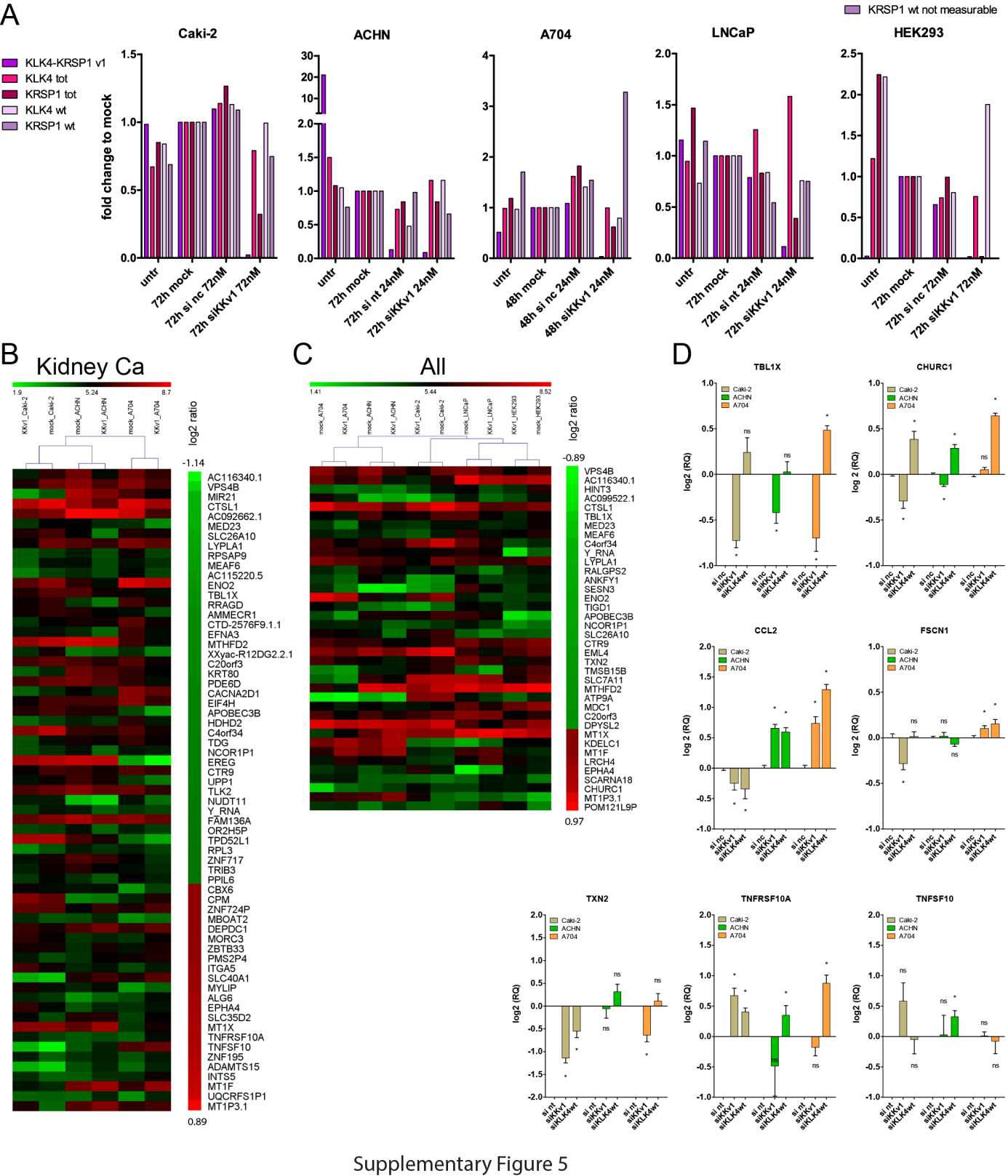
**

**Supplementary Figure 6:**

**(A)** *IL8* expression in frozen RCC Tumor and matched Normal tissues measured by quantitative PCR (qPCR) using TaqMan plotted as mean ± SEM. **(B)** *LITAF* expression in frozen RCC tissues, with or without *KKv1* expression, plotted as mean ± SEM. Significance was calculated using student’s t-test (* p<0.05, ns=not significant)


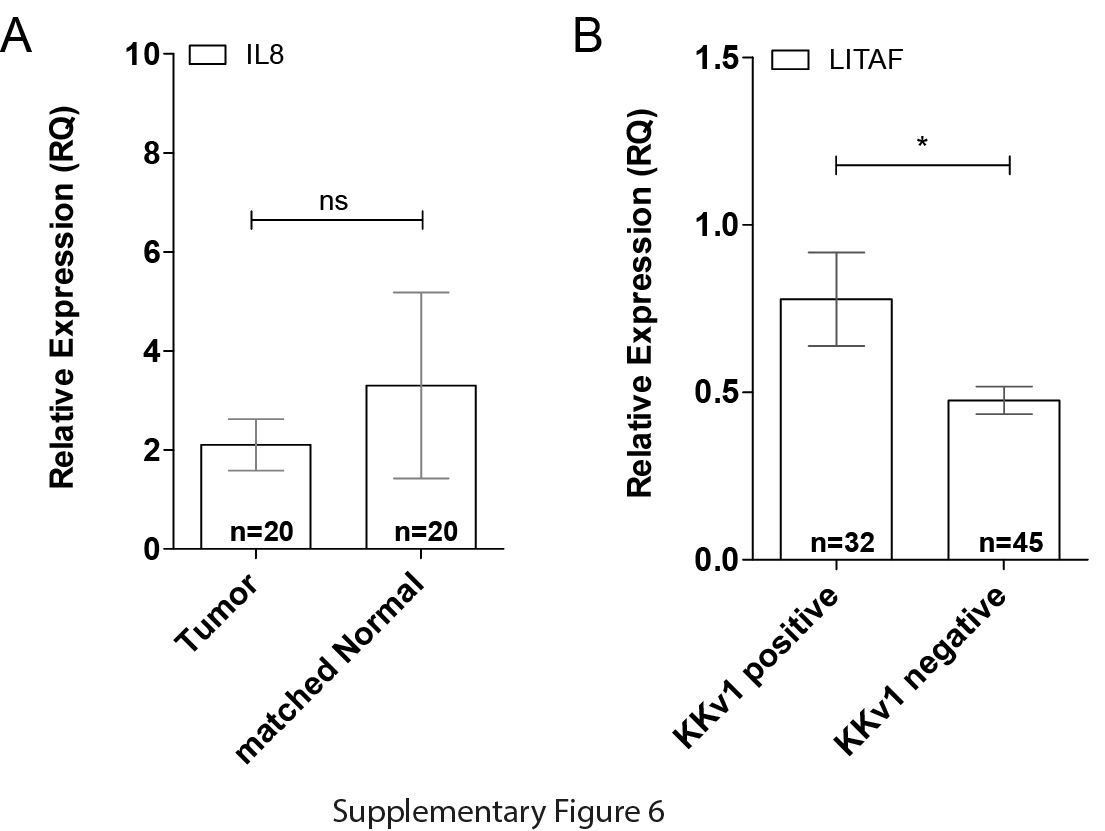


**Supplementary Figure 7:**

**(A)** Knock-down verification of 3 cell lines subjected to migration/invasion experiments. * p<0.05 and ** p<0.01 for *BGv1* up-regulation upon si*GATMwt* in ACHN and Caki-2 as calculated by student’s t-test. **(B)** Representative images of the migratory behavior of Caki-2, ACHN and A704 cells under *BG* and *GATMwt* knock-down using the xCELLigence RTCA DP system where the cells migrate from a chemoattractant-poor towards a chemoattractant-rich environment through a porous membrane equipped with electrodes measuring an increase in impedance as cells migrate through the pores. Plotted are these impedance changes (Delta cell index, representing the number of migrated cells) over time. The slopes of such curves where calculated, normalized to si nc and plotted as bar charts in **Figure 7**. si*BG* and si*GATMwt* are shown in separate images in order to be presented with their respective si nc control. The migration of cells without any addition of chemoattractant serves as negative control. **(C)** Representative images of migration of Caki-2, ACHN and A704 subjected to *KKv1* and *KLK4wt* knock-down.


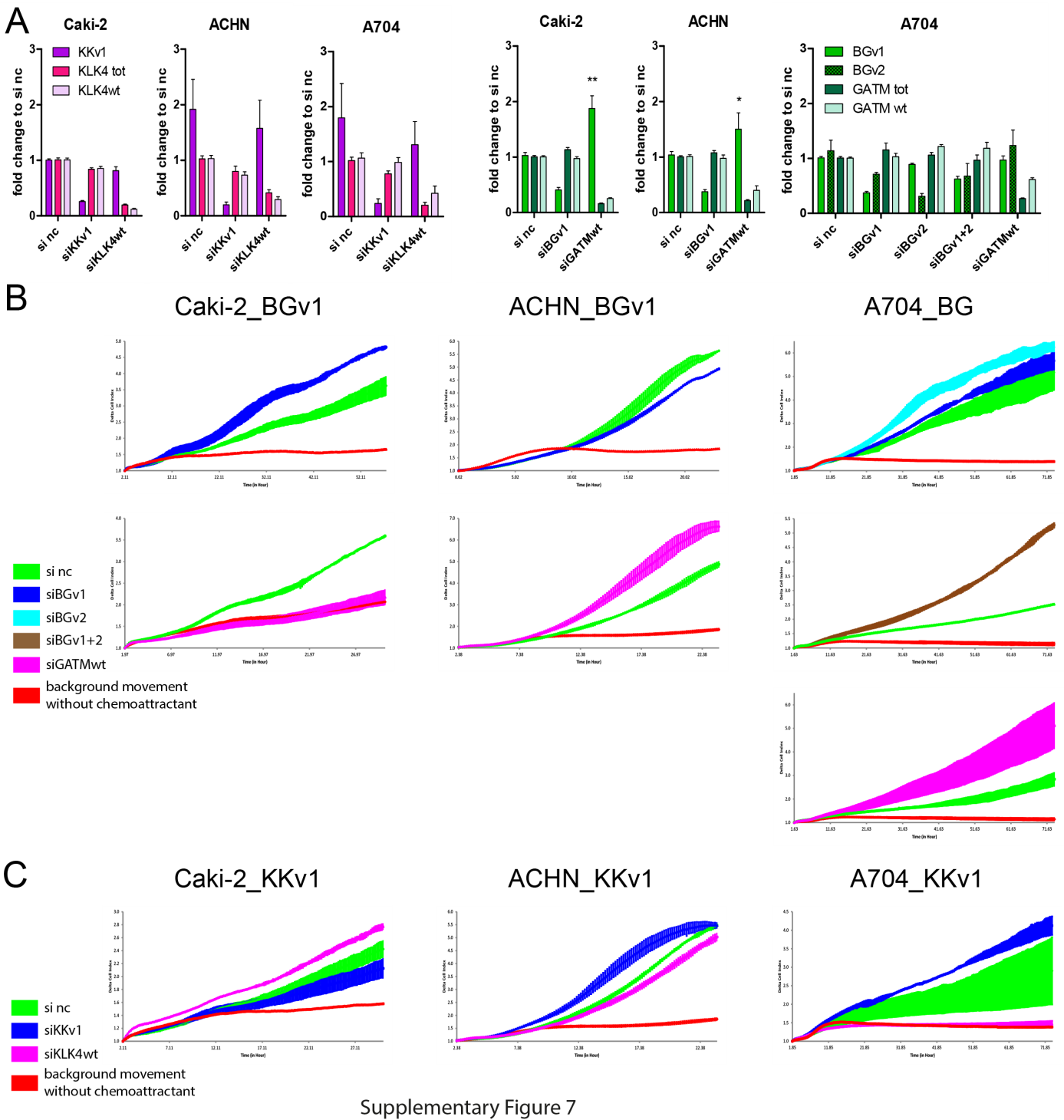


**Supplementary Figure 8:**

**(A**) Knock-down verification of 5 cell lines subjected to proliferation experiments. **(B)** Measurement of proliferation rate using BrdU assays in 5 cell lines subjected to read-through knock-downs for 48h and 72h as indicated. Plotted are the mean ± SEM of n biological replicates as indicated. The only significant change was observed for 48h si*BGv1* in HK-2 as indicated. **(C)** Plot of cell numbers counted from the cells used in (**B**) after read-through knock-down for 48h and 72h as indicated. **(D)** Measurement of viability/metabolic rate using MTT assays in 5 cell lines subjected to read-through knock-downs for 48h and 72h as indicated.


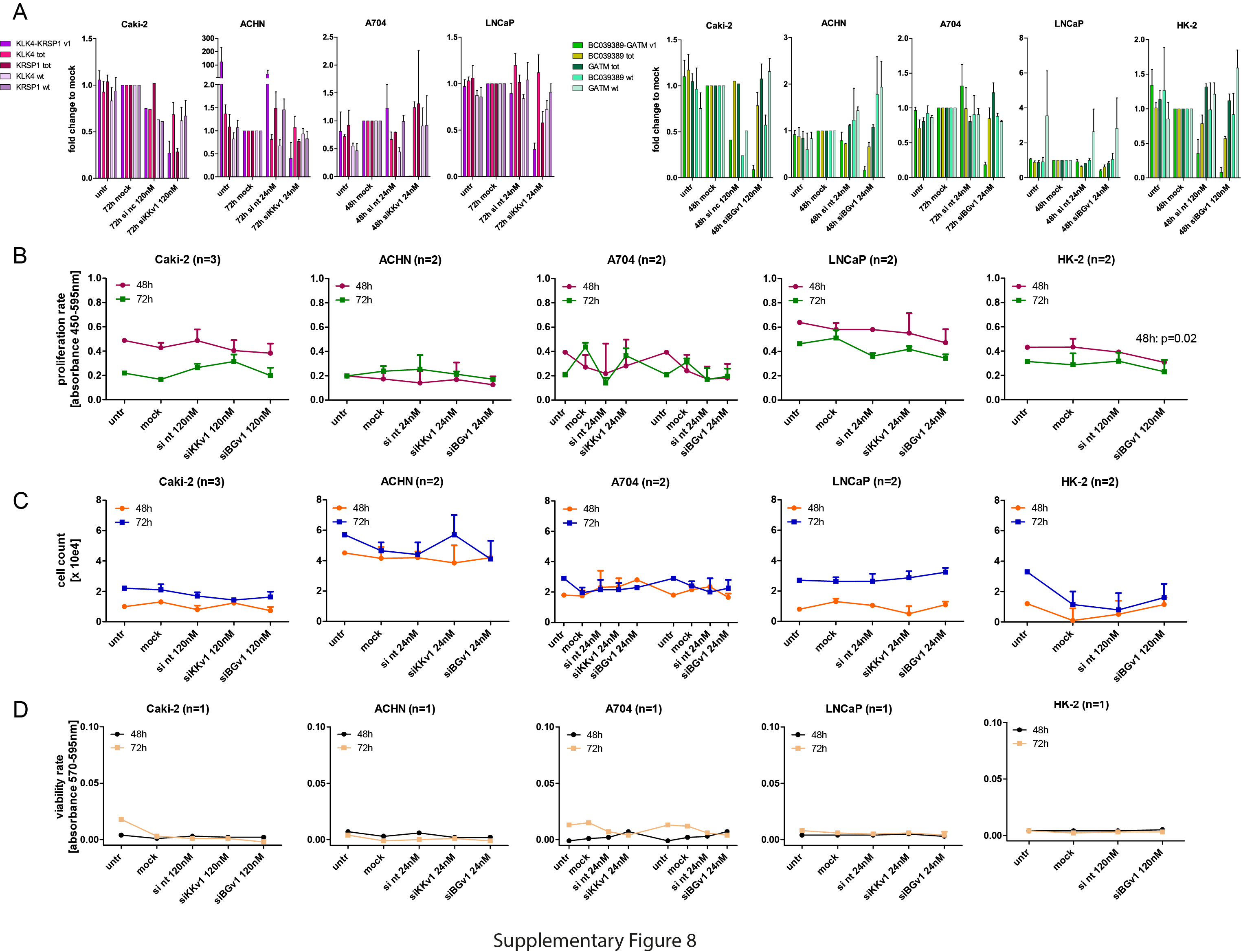


**Supplementary Figure 9:**

**(A)** Both *BG* isoforms encode the same ORF of N-terminally truncated GATM. GATM consists of 5 modules (m) indicated in pink, purple, green, yellow and orange; the first amino acid (aa) number of each module is noted on top of the box. Critical aa for GATM wild-type protein function are based on GATM crystal structure [[3](#_ENREF_3)] and indicated in blue together with their aa positions. Amino acids of the catalytic triad are indicated in red. The first 5 aa of the proteins are named in the box representing module 1. BG protein starts at aa 130 of the corresponding wild-type GATM, indicated by the dashed grey line. The GATM antibody is binding both protein forms within a region indicated by an orange roundish rectangle. The molecular weight of the proteins (including the C-terminal 1xHA-tag) is given behind their schemata. **(B)** Western Blot showing GATMwt and putative BG levels in a panel of cell lines. **(C)** Western Blot showing GATMwt and putative BG levels in matching pairs of tumor (T)/normal (N) kidney tissue from 6 RCC patients. **(D)** Immunoprecipitation (IP) of GATMwt and putative BG from ACHN and A704 whole cell lysate. We can assume that GATMwt (48kDa) is partly obscured from IgG heavy chain (approx. 50 kDa). IP of ACHN is shown with various combinations of lysis and wash buffers for the sake of optimization. A second elution from the same IP columns showing the strongest precipitation of the putative BG band was loaded on the Western Blot. The resulting bands from IPs were compared in size to exogenously over-expressed BG and GATMwt in HEK293T cells. **(E)** IP in ACHN and A704 using an isotype IgG as negative control. **(F)** Before and after comparison of a Commassie-stained gel loaded with IPs of ACHN using isotype IgG and anti-GATM antibody. Shown are the upper (u) and lower (l) part of a band and the height of putative BG that was cut and subjected to mass spectrometry. **(G-H)** A time series of triple knock-down of both *BG* isoforms together with the total transcripts of *GATM* (represented by exon 7) in ACHN (**G**) and HK-2 (**H**) does not evoke any reduction of the putative BG band. GATMwt could be successfully knocked-down time-dependently.

**
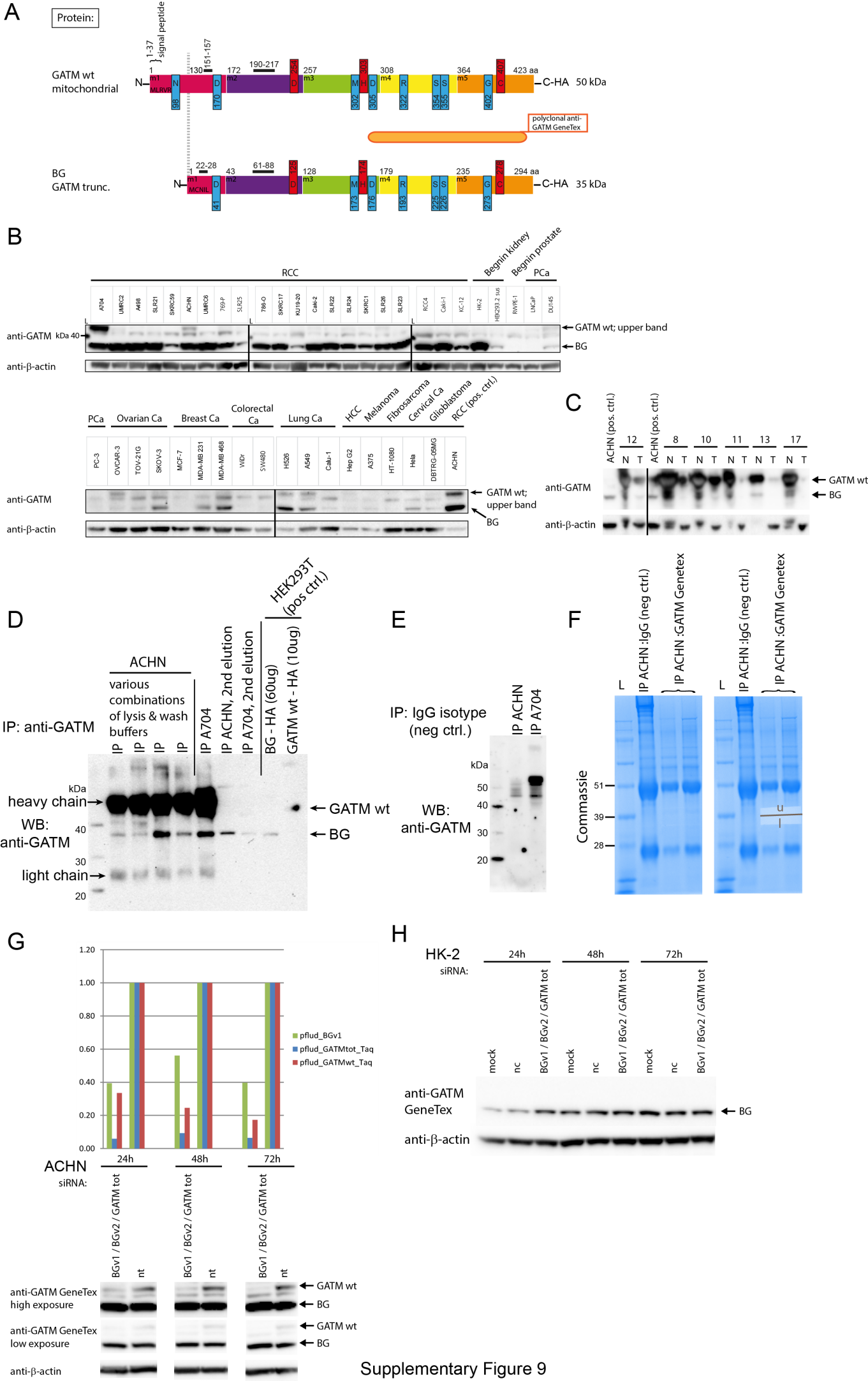
**

**Supplementary Figure 10:**

**(A)** The proteins encoded by each RNA isoform of *KLK4-KRSP1* is given and aligned with the long and short (alternative) KLK4 protein. Amino acids of the catalytic triad are based on KLK4 crystal structure [[4](#_ENREF_4)] and indicated in red. Pre-pro-peptides that are cleaved off for zymogen activation are indicated as orange curly braces. The grey lines indicate different sections of alignment, not functional units. The first and last four amino acids are given for each section along with the aa count at the start and the end of the sections. Total aa numbers are at the end of each protein variant. The molecular weight of the protein isoforms is given below their names, including the weight of the C-terminal 1xHA-tag for the ones chosen to be cloned for exogenous expression in cell lines (indicated by a red asterisk). The KLK4 antibody is binding within a region indicated by an orange roundish rectangle. **(B-C)** Transient over-expression of KK ORFs in HEK293T (**B**) and ACHN (**C**). KKv1 alt ORF is over-expressed at low levels in HEK293T but can also be detected in ACHN with more intense exposure. **(D)** RNA levels in LNCaP (a known androgen-responsive PCa cell line) are peaking at 12h hormone exposure with Dihydrotestosterone (DHT). The effect is abrogated upon the addition of MG132 which is reducing the RNA levels. Optimal concentrations and time points were tested in pre-experiments (not shown) **(E)** RNA levels in Caki-2 are not increased upon hormone stimulation alone. Only with addition of MG132, the RNA levels increased. **(F-G)** KK protein translation is not induced upon treatment with hormone DHT and/or proteasome inhibitor MG132 in LNCaP (**F**) or Caki-2 (**G**). MCL1 is a positive control for blockage of proteasomal protein degradation. CANT1 is used as positive control for hormone stimulation. ORFs over-expressed in HEK293T were loaded as positive controls. **(H)** *KKv1* levels in UOK146, an Xp11 translocation RCC cell line, in comparison to Caki-2 and LNCaP. **(I)** Immunoprecipitation (IP) of putative endogenous KKv1 from UOK146. No precipitated band appears at the expected size of KKv1. The KLK4 antibody proofs functional and specific in the precipitation of KKv1 and KLK4wt exogenously over-expressed in HEK293T.


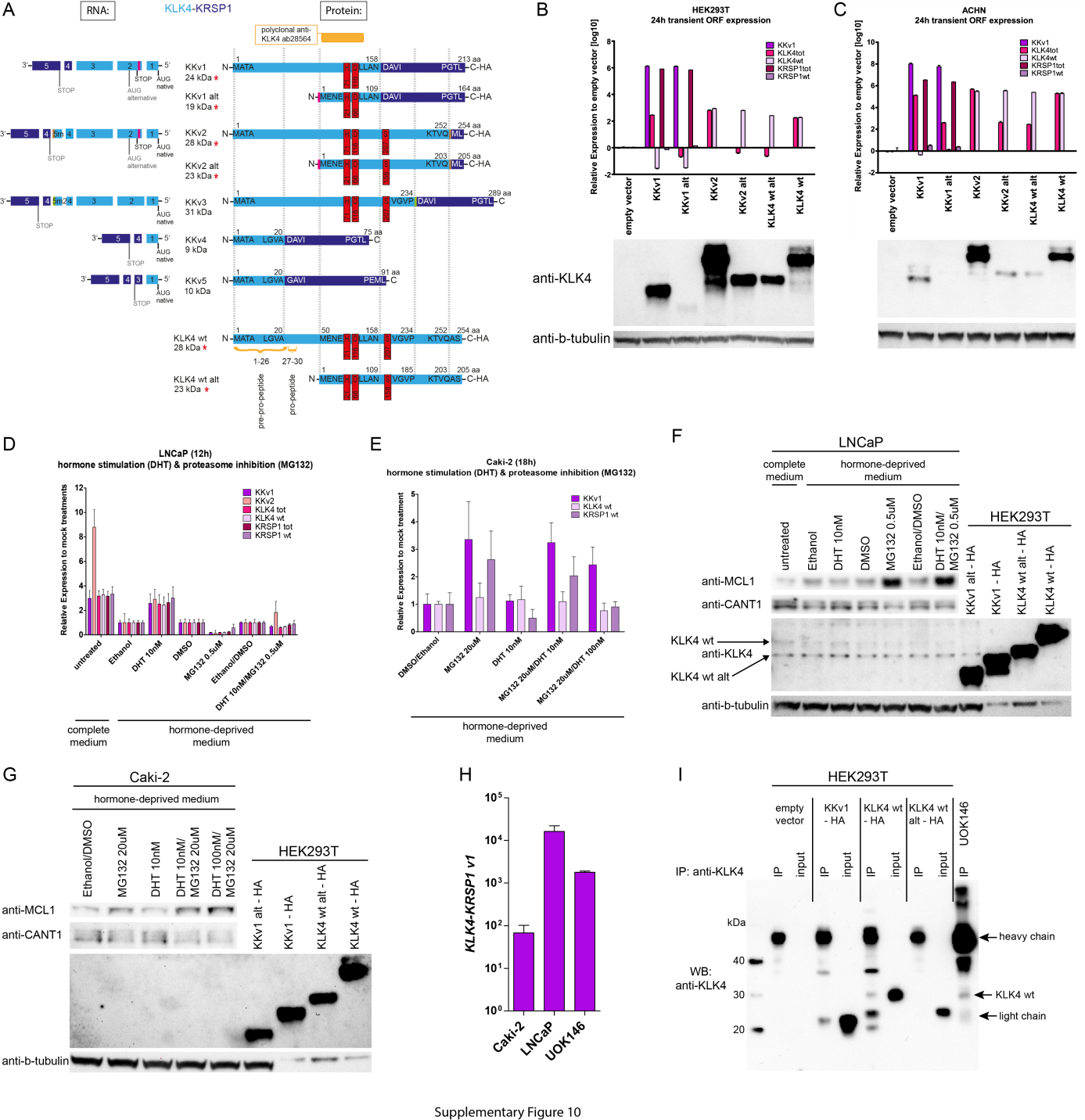


**Supplementary Figure 11:**

Expression of **(A)** *KKv1* and **(B)** *BGv1* in the nuclear and cytoplasmic compartment of three cell lines.


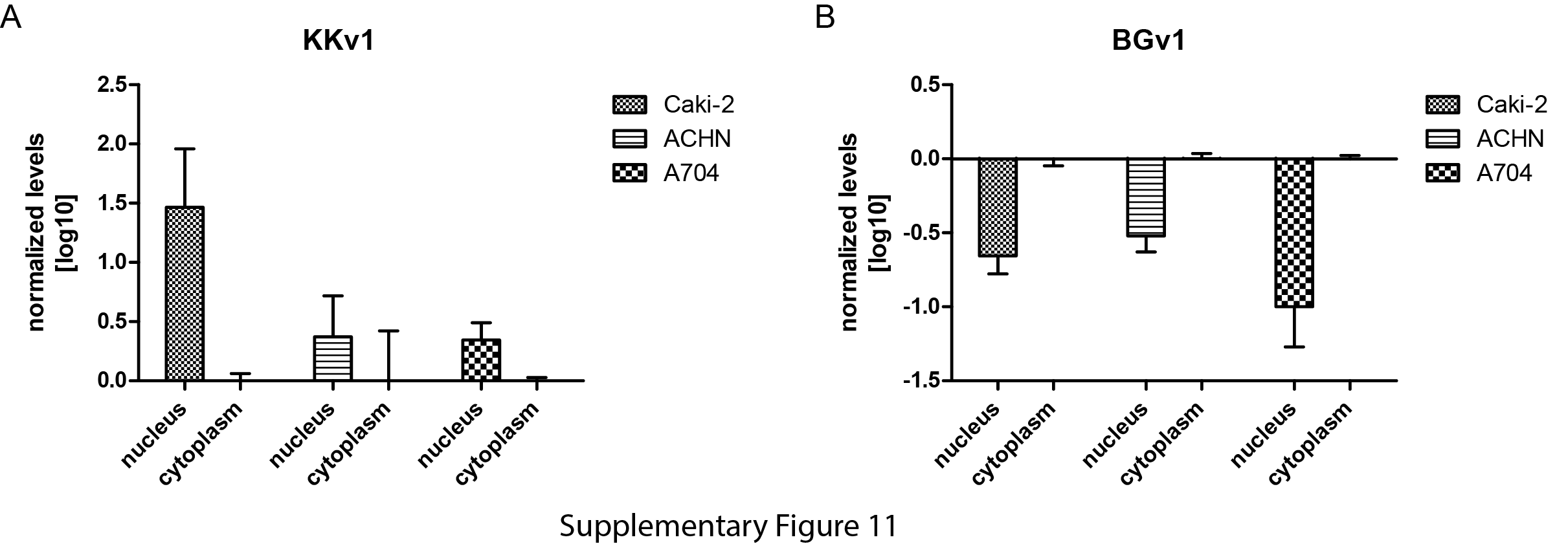


**References**

1. Korkmaz KS, Korkmaz CG, Pretlow TG, Saatcioglu F: **Distinctly different gene structure of KLK4/KLK-L1/prostase/ARM1 compared with other members of the kallikrein family: intracellular localization, alternative cDNA forms, and Regulation by multiple hormones**. *DNA Cell Biol* 2001, **20**(7):435-445.

2. Zaghlool A, Ameur A, Nyberg L, Halvardson J, Grabherr M, Cavelier L, Feuk L: **Efficient cellular fractionation improves RNA sequencing analysis of mature and nascent transcripts from human tissues**. *BMC biotechnology* 2013, **13**:99.

3. Humm A, Fritsche E, Steinbacher S, Huber R: **Crystal structure and mechanism of human L-arginine:glycine amidinotransferase: a mitochondrial enzyme involved in creatine biosynthesis**. *EMBO J* 1997, **16**(12):3373-3385.

4. Debela M, Magdolen V, Grimminger V, Sommerhoff C, Messerschmidt A, Huber R, Friedrich R, Bode W, Goettig P: **Crystal structures of human tissue kallikrein 4: activity modulation by a specific zinc binding site**. *J Mol Biol* 2006, **362**(5):1094-1107.
